# ISG20L2: an RNA nuclease regulating T cell activation

**DOI:** 10.1007/s00018-023-04925-2

**Published:** 2023-08-30

**Authors:** Ana Rodríguez-Galán, Sara G. Dosil, Anna Hrčková, Lola Fernández-Messina, Zuzana Feketová, Julie Pokorná, Irene Fernández-Delgado, Emilio Camafeita, Manuel José Gómez, Marta Ramírez-Huesca, Cristina Gutiérrez-Vázquez, Fátima Sánchez-Cabo, Jesús Vázquez, Štěpánka Vaňáčová, Francisco Sánchez-Madrid

**Affiliations:** 1 Instituto Investigación Sanitaria Princesa (IIS-IP), Immunology Service, Hospital Universitario de la Princesa, Universidad Autónoma de Madrid (UAM), Madrid, Spain; 2 Intercellular Communication in the Inflammatory Response, Vascular Pathophysiology Area, Centro Nacional de Investigaciones Cardiovasculares (CNIC), Madrid, Spain; 3 Universidad Autónoma de Madrid, Madrid, Spain; 4 CEITEC—Central European Institute of Technology, Masaryk University, Kamenice 5/A35, 625 00 Brno, Czech Republic; 5 Faculty of Medicine, Masaryk University, Kamenice 5, 625 00 Brno, Czech Republic; 6 Centro de Investigación Biomédica en Red, Enfermedades Cardiovasculares (CIBERCV), Madrid, Spain; 7 Universidad Complutense de Madrid, Madrid, Spain; 8 Cardiovascular Proteomics Laboratory, Centro Nacional de Investigaciones Cardiovasculares (CNIC), Madrid, Spain; 9 Bioinformatics Unit, Centro Nacional de Investigaciones Cardiovasculares (CNIC), Madrid, Spain

**Keywords:** ISG20L2, Exonuclease, T cell, Immunoregulatory

## Abstract

ISG20L2, a 3′ to 5′ exoribonuclease previously associated with ribosome biogenesis, is identified here in activated T cells as an enzyme with a preferential affinity for uridylated miRNA substrates. This enzyme is upregulated in T lymphocytes upon TCR and IFN type I stimulation and appears to be involved in regulating T cell function. ISG20L2 silencing leads to an increased basal expression of CD69 and induces greater IL2 secretion. However, ISG20L2 absence impairs CD25 upregulation, CD3 synaptic accumulation and MTOC translocation towards the antigen-presenting cell during immune synapsis. Remarkably, ISG20L2 controls the expression of immunoregulatory molecules, such as AHR, NKG2D, CTLA-4, CD137, TIM-3, PD-L1 or PD-1, which show increased levels in ISG20L2 knockout T cells. The dysregulation observed in these key molecules for T cell responses support a role for this exonuclease as a novel RNA-based regulator of T cell function.

**Supplementary Information:**

The online version contains supplementary material available at 10.1007/s00018-023-04925-2.

## Introduction

T cell activation is a tightly regulated process which is dynamically controlled by the balance of a plethora of activating and inhibitory cell surface membrane receptors and cytosolic molecules including adapters, kinases, phosphatases, and other enzymes [1, 2]. MiRNAs are among the molecules whose repertoire undergoes an intense remodelling upon activation. These small (~ 19–24 nucleotides) single-stranded non-coding RNA molecules act as post-transcriptional regulators controlling gene expression, either by promoting mRNA degradation or repressing their translation [3]. The global miRNA decay occurring in T cells only few hours after stimulation [4, 5], together with a specific association of activation-driven downregulated miRNAs with uridylation [6, 7], point to the existence of an active degradation mechanism that would target preferentially miRNAs bearing 3′ uridines. A number of enzymes, including Dis3L2 and Eri1, may account for this intense and rapid miRNA degradation. Dis3L2 has been described to degrade uridylated precursor and mature miRNAs [8–13]. Eri1 is another 3′ to 5′ exonuclease which degrades oligouridylated histone mRNA [14] and reduces miRNA levels in CD4 + T cells and NK cells [15]. Thus, Dis3L2 and Eri1 may account, at least partially, for miRNA decay after T cell activation.

To search for novel exonucleases responsible for degrading uridylated miRNAs, we designed a pull-down strategy using modified miRNAs with different 3′ tails as bait. As a result, we identified ISG20L2, a new potential regulator of T cell responses, which could be controlling T cell activation balance by regulating the expression of immunoregulatory molecules.

## Materials and methods

### Human primary CD4 + T cell isolation and activation

Human peripheral blood mononuclear cells (PBMCs) were isolated from buffy coats, obtained from healthy donors, by separation on Biocoll Separating Solution (Biochrom, L6115), according to standard procedures. Non-adherent cells were separated from PBMCs after a 30 min adherence step at 37 °C. Next, CD4 + T cells were purified from remaining non-adherent cells using the Human Resting CD4 + T cell Isolation Kit (STEMCELL Technologies, 17962) and following manufacturer´s instructions. For T cell stimulation, we treated CD4 + T cells with either αCD3αCD28 (ImmunoCult™ Human CD3/CD28 T Cell Activator; STEMCELL Technologies, 10971) or IFN I (1:1000, Human IFN Alpha Hybrid (Universal Type I IFN); PBL ASSAY SCIENCE, 11200-1). Cells were cultured in RPMI 1640 (Gibco), supplemented with 10% fetal bovine serum (FBS, Sigma), 20 mM Hepes (Hyclone), 0.3 mg/mL L-glutamine (Hyclone), 100 U/mL penicillin (Gibco) and 100 μg/mL streptomycin (Gibco).

For nucleofection experiments, total human CD4 + T cells were isolated with EasySep Human CD4 + T Cell Isolation Kit (STEMCELL Technologies, 17,952) and cultured in X-VIVO 15 (Lonza). Cells were stimulated with αCD3αCD28 (ImmunoCult™ Human CD3/CD28 T Cell Activator; STEMCELL Technologies, 10971).

### Human lymphoid cell lines culture and activation

Jurkat-derived T cell line J77 E6.1 (Vαl.2 Vβ8 + TCR) and lymphoblastoid B cell line Raji were obtained from ATCC. These human cell lines were cultured in the same media used for primary cells and routinely tested for potential mycoplasm contamination.

J77 cells were activated (in the RPMI supplemented media used for primary cells, but containing only 5% FBS) either with: αCD3αCD28 (αCD3 5 μg/mL, αCD28 2 μg/mL Figs. 4C, 6F), αCD3αCD28 (20μL/mL ImmunoCult™ Human CD3/CD28 T Cell Activator; STEMCELL Technologies, 10971 Fig. 6F), PMA (phorbol 12-myristate 13-acetate, 5 ng/mL; Sigma-Aldrich, p-8139) and ionomycin (250 ng/mL; Sigma-Aldrich, I0634) (Figs. 5F, G, 6F, SupFig.4), or with Raji B cells and SEE (Figs. 5A–E, 6A–E). In the latter approach, Raji B cells were incubated with SEE (0.5 μg/mL; Toxin Technologies, PE404), for 30 min at 37 °C. Then, SEE pulsed Raji B cells were washed and cultured with J77 T cells. For flow cytometry assays 150000 cells J77 and 150 000 Raji cells were plated in P96 wells. Plates were centrifuged at low speed to favour earlier proximity between cells and incubated at 37 °C. Co-cultures without SEE were prepared in parallel as controls.

### CRISPR-Cas9 plasmids

CRISPR-Cas9 plasmids including sgRNA targeting human ISG20L2 were obtained following the protocol described by Ran et al. [16]. Plasmid pSpCas9(BB)-2A-GFP (PX458) (available from AddGene) was modified to incorporate specific sgRNA (single guide RNA). Target sequences were selected using CHOPCHOP [17, 18] (http://chopchop.cbu.uib.no). For each target sequence, a pair of oligos was designed including an overlapping region with the plasmid, followed by G (if the target sequence does not present a G on 5′) to facilitate transcription [Target1 (Fwd: CACCGTTGTGGACTACCGAACCAGG, Rev: AAACCCTGGTTCGGTAGTCCACAAC), Target6 (Fwd: CACCGGGAGCTCTTCTTCTGAGAG, Rev: AAACCTCTCAGAAGAAGAGCTCCC), Target8 (Fwd: CACCGAGAAGCGGAGGCTCTTAGAA, Rev: AAACTTCTAAGAGCCTCCGCTTCTC)]. Oligos were phosphorylated (T4 Polynucleotide Kinase; Thermo Scientific, EK0031) and annealed, and plasmid digested (FastDigest *Bbs*I; Fermentas/Thermo Scientific, FD1014); prior to the ligation reaction (T4 DNA ligase; Promega, M1801). Finally, plasmids purified from DH5α transformed with the ligation product and cultured under ampicillin selection, were sequenced to confirm the specific sgRNA incorporation.

### Cell transfection

Prior to electroporation, J77 cells were washed first with PBS or HBSS, and afterwards with Opti-MEM (Gibco). Cells were then resuspended in Opti-MEM at 30 × 10^6^ cells/mL and 400 μL (12 × 10^6^ cells/mL) were added to each 4 mm Bio-Rad cuvette together with the corresponding plasmids (a total of 10 μg of DNA). Cells were electroporated with a Gene Pulser Xcell Electroporation System (Bio-Rad) at 240 mV and 975 μF. After electroporation, J77 cells were cultured in medium containing 5% FBS. Dead cells were discarded using Biocoll Separating Solution (Biochrom, L6115) and GFP + cells were isolated by FACS sorting.

Human CD4 + T cells isolated from healthy donor’s buffy coats and activated for 2 or 3 days were nucleofected with CRISPR-Cas9 plasmids using Amaxa Human T cell Nucleofector Kit (Lonza, VPA-1002) and Amaxa Nucleofector II (Lonza). In each cuvette, a total of 5–6 × 10^6^ pre-activated cells (2–3 days with ImmunoCult™, STEMCELL Technologies, 10971) and 1–5 μg of DNA were placed and T-020 programme was set. Nucleofected cells were rested overnight, sorted to isolate GFP + cells and αCD3αCD28 re-activated. Pre- and post-nucleofection human CD4 + T cells were cultured in X-VIVO 15 (Lonza).

### ISG20L2 KO clones

J77 cells were transfected with a combination of two plasmids with sgRNA directed towards two different targets (1 + 6 or 6 + 8). One GFP + cell was sorted to each well (96-well plate), containing pre-conditioned media. This media was obtained from a J77 cell culture after centrifuging at high speed, and filtering the supernatant with a 0.22 µm strainer. For genotyping forward (CCTCTTTTCATCCATAAGCCAC) and reverse (TAGACCTCTCTCCATCCACCTC) were designed to amplify by PCR a region (856nt) including all targets (1, 6 and 8). Knock out clones were identified for removal of fragments 1–6 (274nt) or 6–8 (302nt), leading to an amplification of a smaller region (582 and 554, respectively). Genomic DNA was isolated with Gentra Puregene cell kit (Qiagen, 158745-K). PCR was performed with REDExtract-N-Amp PCR Reaction mix (Sigma-Aldrich, R4775), and resolved in 3% agarose (Agarose D1 Low EEO, Covalab) gels stained with ethidium bromide (Sigma-Aldrich, E-1510).

### RNAseq and smallRNAseq in J77 cells (control and ISG20L2 knockout)

Three independent experiments were performed electroporating J77 cells with pSpCas9(BB)-2A-GFP (control cells), or pSpCas9(BB)-2A-GFP-sgRNA_target1 and pSpCas9(BB)-2A-GFP-sgRNA_target6 (knockout cells). After 72 h, GFP + cells isolated by FACS sorting were lysed in QIAzol Lysis Reagent (Qiagen, 79306) and RNA was extracted using the miRNeasy Mini Kit (Qiagen, 217004). In order to reduce phenol-based reagent contaminations, purified RNA samples were precipitated using sodium acetate (3 M, 0.1 × sample volume) and ethanol (100%, 3 × sample volume). RNA integrity was evaluated using an Agilent 2100 Bioanalyzer (Eukaryote Total RNA Nano assay).

For smallRNAseq, 100 ng of total RNA (not limited to the miRNA size in order to include also other small RNAs) were used to generate barcoded smallRNA-seq libraries using the NEBNext Multiplex SmallRNA Library Prep Set for Illumina (New England Biolabs). Briefly, 3′ and 5′ SR adapters were first ligated to the RNA sample. Next, reverse transcription followed by PCR amplification was used to enrich cDNA fragments with adapters at both ends. The quantity and quality of the smallRNA libraries were determined using the Agilent 2100 Bioanalyzer High Sensitivity DNA chip. Libraries were sequenced on a HiSeq2500 (Illumina) to generate 60 bases single reads. FastQ files for each sample were obtained using bcltofastQ 2.20 Software software (Illumina). NGS experiments were performed at the Genomics Unit of the CNIC.

For mRNAseq, 100 ng of total RNA were used to generate barcoded RNA-seq libraries using the NEBNext Ultra II Directional RNA Library preparation kit (New England Biolabs) according to manufacturer’s instructions. First, poly A + RNA was purified using poly-T oligo-attached magnetic beads followed by fragmentation and first and second cDNA strand synthesis. Next, cDNA ends were repaired and adenylated. The NEBNext adaptor was then ligated followed by second strand removal, uracile excision from the adaptor and PCR amplification. The size of the libraries was checked using the Agilent 2100 Bioanalyzer and the concentration was determined using the Qubit^®^ fluorometer (ThermoFisher). Libraries were sequenced on a HiSeq2500 (Illumina) to generate 60 bases single reads. FastQ files for each sample were obtained using CASAVA v1.8 software (Illumina).

SmallRNAseq reads were pre-processed by means of a pipeline that used FastQC (http://www.bioinformatics.babraham.ac.uk/projects/fastqc/) to assess read quality; and Cutadapt [19] to trim sequencing reads, eliminating Illumina adaptor remains, and to discard those that were shorter than 15 nt after trimming. Around 34% of the reads from any of the samples were retained. Resulting smallRNAseq reads were aligned against a collection of 2657 human, mature miRNA sequences extracted from miRBase (release 22), to obtain expression estimates with RSEM [20]. Percentages of reads participating in at least one reported alignment were around 47%. Expected expression counts were then processed with an analysis pipeline that used Bioconductor package Limma [21] for normalization (using TMM method) and differential expression testing, taking into account that samples had been processed as three pairs, and considering only 597 miRNA species for which expression was at least 1 count per million (CPM) in 3 samples. Changes in gene expression were considered significant if associated to Benjamini–Hochberg adjusted *p*-value < 0.1.

sRNAtoolbox (https://arn.ugr.es/srnatoolbox/) was used as a complementary system to quantify the abundance of the different types of small RNA detected.

RNAseq data analysis was performed following the steps described for small RNAseq with the following variations. After trimming, discarded sequences were those shorter than 30nt. Reads were mapped against reference transcriptome GRCh38.91. Percentages of reads participating in at least one reported alignment were around 84%.

Core analysis were performed by Ingenuity Pathway Analysis (Content version: 49932394 (Release Date: 2019-11-14).

### Immunoblotting

Cell extracts were prepared in lysis buffer (50 mM Tris pH 7.5, 150 mM NaCl, 1%NP-40, 5 mM EDTA, 50 mM NaF, 5 mM DTT) supplemented with a protease inhibitor cocktail (Complete, Roche). Cell lysates were cleared of debris and nuclei by centrifugation (15000 *g*, 15 min), mixed with Laemli solution and β-mercaptoethanol (2% final volume) and boiled 5 min at 90 °C. Proteins were separated on 8–10% SDS-PAGE gels and transferred to a nitrocellulose membrane. Membranes were incubated with primary specific antibodies and peroxidase conjugated secondary antibodies. Chemoluminescence was measured with LAS-3000 (Fujifilm). Band intensities were quantified with Image Studio Lite (LI-COR Biosciences), normalized to ERMs values and relativized to unstimulated conditions (when no band was detected at 0 h, background was taken as reference signal).

### MiRNA pull-down

Streptavidin dynabeads (Dynabeads MyOne Streptavidin C1; Invitrogen, 65001) were loaded with biotinylated miRNAs according to manufacturer instructions. Per condition, 12–20 × 10^6^ J77 cells were lysed in 1 mL of buffer [Lysis buffer: PBS, NP40 1%, NaCl 150 mM, EDTA 5 mM, NaF 50 mM, DTT 5 mM, Complete (Roche Diagnostics, 11836145001) and PhosSTOP (Roche, 4906837001)]; and after nuclei and debris clearance, incubated overnight with beads (50 μL). UV-Crosslink (400 × 100 mJ; Stratalinker UV crosslinker, Stratagene) was performed and beads were extensively washed (6–7 times) [Washing buffer: PBS, NP40 0.05%, NaCl 150 mM, EDTA 5 mM, NaF 50 mM, DTT 5 mM and Complete]. Finally, beads were prepared in 20μL of washing buffer for LC–MS/MS or boiled in Laemmli for western blot analysis. For ISG20L2-GFP pull-down validation, lysed J77 cells had been previously electroporated with pEGFP-C1-ISG20L2.

### LC–MS/MS analysis and peptide identification

Proteins bound to the beads were subjected to one-step in-gel tryptic digestion as described previously [22]. The dried peptides were taken up in 0.1% (v/v) formic acid and loaded onto a PepMap100 C18 LC pre-column (75 μm I.D., 2 cm, Thermo Scientific) and eluted on line onto an analytical NanoViper PepMap100 C18 LC column (75 μm I.D., 50 cm, Thermo Scientific) with a continuous gradient consisting of 8–30% B in 90 min (B = 80% ACN, 0.1% formic acid) at 200 nL/min, using an Easy nLC-1200 nano-HPLC (Thermo Scientific, San Jose, CA, USA) coupled to a hybrid quadrupole-orbitrap mass spectrometer (Q Exactive HF, Thermo Scientific). Peptides were ionized using a Picotip emitter nanospray needle (New Objective). Each MS run consisted of an enhanced FT-resolution spectra (120000 resolution) in the 400–1200 m/z range followed by data-dependent MS/MS spectra of the 20 most intense parent ions acquired along the chromatographic run. The AGC target value in the Orbitrap for the survey scan was set to 1000000. Fragmentation in the linear ion trap was performed at 30% normalized collision energy with a target value of 10000 ions. The full target was set to 30000, with 1 microscan and 50 ms injection time, and the dynamic exclusion was set to 0.5 min.

For peptide identification the MS/MS spectra were searched with the Sequest algorithm implemented in Proteome Discoverer 1.4 (Thermo Scientific). Database searching against human protein sequences from the UniProt database (March 2017, 158382 entries) was performed with the following parameters: trypsin digestion with 2 maximum missed cleavage sites; precursor and fragment mass tolerances of 800 ppm and 0.02 Da, respectively; Cys carbamidomethylation as a static modification; and Met oxidation as a dynamic modification. The results were analysed by the probability ratio method [23] using 15 ppm precursor postfiltering and a 0.01 false discovery rate (FDR) threshold for peptide identification [24], based on the search results against a decoy database using the refined method [25].

### Recombinant ISG20L2 expression and purification

In order to prepare the ISG20L2 catalytic mutant, critical positions in the active site were hypothesized based on homology with two closely related DEDDh enzymes, ISG20 and ISG20L1 (AEN). The three enzymes were aligned using COBALT (Constraint-based Multiple Alignment Tool) (Supplementary Fig. 1A, B). These proteins share high homology, particularly in the catalytic region (identified by COBALT as DNAQ_like_exo (or DEDD) in ISG20 and ISG20L1; while ISG20L2 catalytic region is recognized as ISG20) (Supplementary Fig. 1A). The conserved aspartate (D) residues are marked in yellow, green and blue following the positions described in UniProt to be involved in metal binding by ISG20 (bold and underlined residues) (Supplementary Fig. 1B). A catalytic mutant of ISG20L2 was generated by site-directed mutagenesis replacing the cysteine in position 184 for a tyrosine (C184Y), by changing the codon TGT to TAT (Supplementary Fig. 1C).

We observed high cellular toxicity for any constructs containing ISG20L2 gene but this was not the case for the catalytically inactive ISG20L2 version. To overcome this issue, we designed a synthetic gene with codon optimized sequence for bacterial expression which was subcloned into pET28. WT and mutant C184Y ISG20L2 were expressed with N-terminal 6xHIS-Smt3 fusion tags in *E. coli* and affinity purified by using NiNTA chromatography. The WT ISG20L2 was expressed at very low levels, as bacterial cells did not survive extended periods of WT protein expression. A large fraction of the protein was deposited in inclusion bodies, resulting in low yield of the purified protein. On the contrary, the C184Y mutant was expressed and purified in sufficient quantities. For the subsequent enzymatic analyses, we concentrated the WT ISG20L2 on Microspin columns.

The tagged WT and C184Y variants were expressed in *E. coli* C43 strain. Several freshly transformed bacterial colonies were inoculated to 5 mL of LB supplemented with 1% glucose, kanamycin (30 mg/L) and chloramphenicol (34 mg/L). Bacterial cultures were grown overnight at 37 °C, harvested by centrifugation, washed twice with sterile PBS, inoculated to 100 mL of autoinduction medium (AIM, Formedium) with kanamycin (30 mg/L) and grown for 15 h at 37 °C. Cells were harvested, pellets resuspended in lysis buffer (50 mM Tris–HCl pH 7.9 at 4 °C, 100 mM KCl, 1 mM PMSF, 10 mM imidazole and 50 µg/mL lysozyme, supplemented with protease inhibitors (cOmplete™, Mini, EDTA-free, Protease Inhibitor Cocktail, Roche) and lysed in homogenizer Microfluidics M110P (18000–20000 psi). Lysate was cleared by centrifugation (10000 g, 45 min, 4 °C) and soluble protein was purified using affinity chromatography on NiNTA- agarose beads. Protein was eluted with elution buffer (50 mM Tris–HCl, pH 7.9 at 4 °C, 10% glycerol, 500 mM KCl, 0.5 mM NP40, 2 mM β-mercaptoethanol, 1.5 mM MgCl_2_ and 300 mM imidazole). The protein samples were concentrated on Vivaspin^®^ 500 with 30000 MWCO and stored at – 80 °C. The purification efficiency was monitored by SDS-PAGE and western blot analyses.

### In vitro RNA degradation assays

RNA miR-151a-3p containing variable 3′ extensions were 5′-terminally labelled using T4 PNK and γ-^32^P ATP, unincorporated nucleotides were removed by using illustra Microspin G-25 columns (GE Healthcare) and RNAs were subsequently purified by denaturing polyacrylamide gel electrophoresis [26, 27].

RNA degradation in vitro assays were performed using two different set ups. In the direct assaying of degradation activity of ISG20L2, 3.5 nM purified recombinant protein was incubated with 25 nM labelled miRNA and 50 mM unlabelled identical RNA in assay buffer (10% glycerol, 25 mM Tris, pH 7.9, 50 mM KCl, 5 mM MgCl_2_, 0.02% NP40), supplemented with 1 mM DTT and RNase inhibitor 0.2 U/µL (Biotechrabbit) at 37 °C for times indicated for particular experiments. In the second arrangement we monitored the ability of specific unlabeled RNAs containing no or different homogeneous nucleotide 3′ extensions to outcompete the isotopically labelled miR-151a-3p + 2Us. In these assays we used 35 nM purified protein, 100 nM labelled RNA and 1 µM unlabelled competing RNA. The reactions were further performed as described above. Reactions were stopped with 1 V of formamide loading buffer (80% formamide, 10 mM EDTA, 100 µg/µL bromophenol blue and 100 µg/µL xylene cyanol) at 95 °C for 5 min. The samples were resolved on 20% polyacrylamide / 8 M urea gel. The signals were detected by autoradiography by using FLA-9000 phosphorimager (Fujifilm). The efficiency of the degradation was quantified by ImageJ as a ratio between the final degradation product and the overall signal in a given lane/sample.

RNA oligonucleotides used for in vitro degradation assays were ordered as synthetic molecules (IDT):

miR-151a-3p: 5′ CUA GAC UGA AGC UCC UUG AGG 3′

miR-151a-3p + 8U: 5′ CUA GAC UGA AGC UCC UUG AGG UUU UUU UU 3′

miR-151a-3p + 8A: 5′ CUA GAC UGA AGC UCC UUG AGG AAA AAA AA 3′

miR-151a-3p + 8G: 5′ CUA GAC UGA AGC UCC UUG AGG GGG GGG GG 3′

miR-151a-3p + 2U: 5′ CUA GAC UGA AGC UCC UUG AGG UU 3′

### Quantitative real-time PCR

For RNA extraction, 100 μL of chloroform (Sigma-Aldrich, C2432) were added to 500μL of QIAzol (Qiagen, 79306) lysed sample, and the mix was shaken vigorously. After 12000 g centrifugation for 15 min at 4 °C, aqueous phase (which contains RNA, while DNA is retained at interphase and proteins at the pink bottom phase) was transferred to a new tube. RNA was precipitated by addition of 250 μL of isopropanol and overnight incubation at – 20 °C. Samples were centrifuged at 12000 g for 10–30 min and supernatants discarded. Pellets were washed with ethanol 75%, centrifuged 7500 g (5 min, 4 °C) and resuspended in RNAse free H_2_O.

Reverse transcription was performed with the High Capacity cDNA Reverse Transcriptase kit (Applied Biosystems, 4,368,814). Quantitative real-time PCR (qPCR) was prepared by triplicate using SYBR green GoTaq qPCR Master Mix (Promega, A6001) and run in a 7900 HT Fast Real-Time PCR system (Life technologies). Data were analysed with SDS2.4 (Applied Biosystems) and QBasePlus (Biogazelle). Expression levels were normalized taking as reference β-actin, B2M, HPRT1 and/or YWHAZ, as indicated. Primers are detailed in the following table:

**Table:** 

Gene	Forward primer	Reverse primer
AHR	ATGGATCAATACTTCCACCTC	TTTGGCATCACAACCAATAG
AQP3	ACCAGCTTTTTGTTTCGGGC	GGCTGTGCCTATGAACTGGT
β-ACTIN	ATCATGTTTGAGACCTTCAA	AGATGGGCACAGTGTGGGT
B2M	GAGGCTATCCAGCGTACTCCA	CGGCAGGCATACTCATCTTTT
BCL2L2	CCTTTGGAATGGAAGCTTAG	GAGGGAATGTTTTCTCCTTG
CD137	ACAACCATTTATGAGACCAG	ACATCCTCCTTCTTCTTCTTC
CD69	CAGCAAAGACTTTCACTGTAG	CATTTTCTTGTCCACTCTCC
CTLA-4	TTGCTAAAGAAAAGAAGCCC	AAAGTTAGAATTGCCTCAGC
DOCK10	CGGAGCCTGTTGAGACCTG	CTAGGCTTTTCTTGCCGCTG
EEF1A1	TATCCACCTTTGGGTCGCTT	GTGGGGTGGCAGGTATTAGG
GITR	AATTCAGTTTTGGCTTCCAG	CAGTCTGTCCAAGGTTTG
H2AFX	GGCCTCCCAGGAGTACTAAGA	CTCTTTCCATGAGGGCGGTG
HPRT1	CCTGGCGTCGTGATTAGTGAT	AGACGTTCAGTCCTGTCCATAA
IDH1	GGCTGTGGTTGTGAGTCTGA	TAGTTTATCGCCTGCCGGG
ISG20L2	GAGATGTGCTTTATGACGAG	TCTTCCCTGTGAGTATCTTC
LAG-3	TATAACCTCACTGTTCTGGG	TCTAGTCGAAGGGTAAAGTC
LEF1	AGAGAGAGAAACTACAGGAATC	CCACCATGTTTCAGATGTAG
NKG2D	AGGACAAAATGACCAAAGAC	CTTGGGGATATCTGAATTGC
PD-L1	ATGCCCCATACAACAAAATC	GACATGTCAGTTCATGTTCAG
PD-1	CTCCAGGCATGCAGATCC	GGCCTGTCTGGGGAGTCTA
RHOH	GCCTTTGCCACTTCTTGGAG	AGCCTAGTCTTCAACTGGTGTG
RPL22L1	TTTTGAGCAATTTCTACGGG	TCAATGTGAACAACATTCCC
TIGIT	GTACTTCTGCATCTATCACAC	GGGCTTTCTTCTTTCTAGTC
TIM-3	CTCTGACTTTTCTTCTGCAAG	ACCTTGTAAGTAGTAGCAGC
TNFAIP3	GCCAAGAGAGATCACACCCC	GCGATCCTTTCGCAAAGTCC
TTLL1	TGGAAAATACCTCTATCTGGACTTT	CCCGGGACCACTTTTTGATCT
TUBA1A	GAAGCAGCAACCATGCGTGA	TCTCCTCCCCCAATGGTCTT
ULK1	GTTCCAAACACCTCGGTCCT	CCAACTTGAGGAGATGGCGT
VISTA	CCCAGGATAGTGAAAACATC	TTCAATCCCTTGAATGTTGC
YWHAZ	AACTTGACATTGTGGACATC	AAAACTATTTGTGGGACAGC
ZNF322P1	GCATTCATTGGAGAGCCTTACT	GGGCCTGATAAGACAGGAGC

### Flow cytometry

Cells were blocked with γ-globulin (100 μg/mL) in PBE (PBS, BSA 0.5%, EDTA 5 mM) at 4 °C for 20 min. Afterwards, cells were stained with corresponding conjugated surface antibodies (1:200, in PBE) at 4 °C for 30 min. Cells were washed with PBS and Propidium Iodide (Sigma-Aldrich, P4864), DAPI (Invitrogen, D3571) or TO-PRO-3 (Invitrogen, T3605) were added to distinguish dead cells.

For intracellular stainings, instead of adding viability dyes at the end, cells were incubated during the blocking step with FYDCS (Fixable Yellow Dead Cell Stain: LIVE/DEAD^®^ Fixable Yellow Dead Cell Stain Kit, for 405 nm excitation; Invitrogen, L34968) in PBS instead of PBE. After surface staining, cells were fixed and permeabilized with FIX&PERM Cell Permeabilization Kit (Invitrogen, GAS-003). Intracellular antibodies were incubated overnight at 1:100 dilution in permeabilization medium.

Proliferation was evaluated staining cells prior to cell culture with CellTrace Violet Cell Proliferation (Invitrogen, C34557).

For Annexin V apoptosis assay, cells were washed twice with PBS and incubated with Annexin V (1:40; BD Pharmingen, 550474) for 15 min in binding buffer (HEPES/NaOH 10 mM, pH 7.4; NaCl 140 mM; CaCl_2_ 2,5 mM).

For IL2 intracellular staining, clones were stimulated 20 h with PMA (5 ng/mL) and ionomycin (250 ng/mL), with the last 3 h of culture using higher PMA and ionomycin concentrations (50 ng/mL and 1 µg/mL, respectively) together with brefeldin A (20 µg/mL, Sigma-Aldrich B7651).

Cells were acquired in a FACS Canto 3L or LSRFortessa (BD Biosciences). Software used for acquisition was BD FACSDiva (BD Biosciences), and samples were analysed with FlowJo (BD Biosciences).

### ELISA

J77 cells were stimulated with PMA (phorbol 12-myristate 13-acetate, 5 ng/mL; Sigma-Aldrich, p-8139) and ionomycin (250 ng/mL; Sigma-Aldrich, I0634), and supernatants were collected either at 20 or 45 h. IL2 detection was performed by ELISA (Human IL2 ELISA Kit, Diaclone, 851500010). Manufacturer’s instructions were followed, but samples were incubated overnight.

### Immunofluorescence

Raji cells were stained with the CMAC cell tracker (10 μM; Molecular Probes, C2110) and pulsed with SEE (0.5 μg/mL; Toxin Technologies, PE404) for 30 min at 37 °C. A total of 150000 Raji cells and 150000 J77 cells were plated on each Poly-L-Lys-coated (50 μg/mL, 1 h, 37 °C) slide, and incubated at 37 °C for 30 min. Cells were fixed with 2% paraformaldehyde in PBS, washed with TBS and permeabilized with 0.2% TritonX-100 (Sigma) in TBS (5 min, RT). Cells were blocked for 30 min (γ-globulin 100 μg/mL, BSA 3%, azide 0.2% in TBS), incubated with primary antibodies (biotinylated CD3 OKT3 and Tubulin-FITC, both 1:100 in blocking buffer), washed, and incubated with stretavidin-647 (Invitrogen, S-21374) and phalloidin conjugated to Alexa Fluor 568 (for actin staining) (both 1:200 in blocking buffer). Slides were mounted on Prolong (Molecular Probes, p36930), and images were captured with a Leica SP5 confocal microscope fitted with a 63 × oil objective.

Maximal projections were assembled using Leica software and analysed with Fiji (ImageJ) (https://imagej.net/Fiji) and Imaris (Oxford Instruments). Accumulation of CD3ε at immune synapse was quantified using ‘Synapse Measures’ plugin for Fiji, developed by Calabia-Linares et al. [28]. Imaris Cell Imaging Software (Oxford Instruments) was used to model Raji B cell surfaces and measure T cell MTOC distance to synaptic contact area [29].

### Live imaging with ColorfulCell reporter plasmid

ColorfulCell reporter plasmid (AddGene,#62449) expresses six fluorescent proteins which allow to visualize cell membrane (Cerulean), nucleus (TagBFP), Golgi apparatus (Citrine), mitochondria (AzamiGreen), endoplasmic reticulum (mCherry) and peroxisomes (iRFP670) [30]. WT and ISG20L2 J77 clones were electroporated with ColorfulCell plasmid as previously indicated in ‘cell transfection’ and incubated overnight. Glass plates (No. 1.5 Mat-Tek Corporation) were coated (1 h at 37 °C or overnight at 4ºC) with antibodies αCD3 (HIT3a, 4.87 μg/mL) and αCD28 (10 μg/mL) diluted in bicarbonate buffer (0.1 M NaHCO_3_, 0.32 M Na_2_CO_3_). Cells were seed onto glass plates covered with HBSS 1.5% FBS 25 mM HEPES. Live imaging was performed (at 37 °C, 5% CO_2_) on a Leica SP8 Navigator confocal laser unit (63 × oil objective). Images were analysed using Imaris Cell Imaging Software (Oxford Instruments).

### Statistical analysis

GraphPad Prism software (La Jolla, CA) was used to perform statistical analysis.

Data were analysed using non parametric tests: Mann–Whitney when comparing two groups and Kruskal–Wallis with Dunn’s multiple comparisons test for more than two groups. Paired samples were studied using Wilcoxon test. Statistical significance is indicated as * (*p*-value < 0.05), ** (*p*-value < 0.01), *** (*p*-value < 0.001) and **** (*p*-value < 0.0001). Relevant *p*-values over 0.05 are indicated with the exact value and ‘ns’ stands for ‘not significant’.

## Results

### ISG20L2, an exonuclease with preferential interaction for uridylated miRNAs, is upregulated in T cell stimulation

**Fig. 1:**
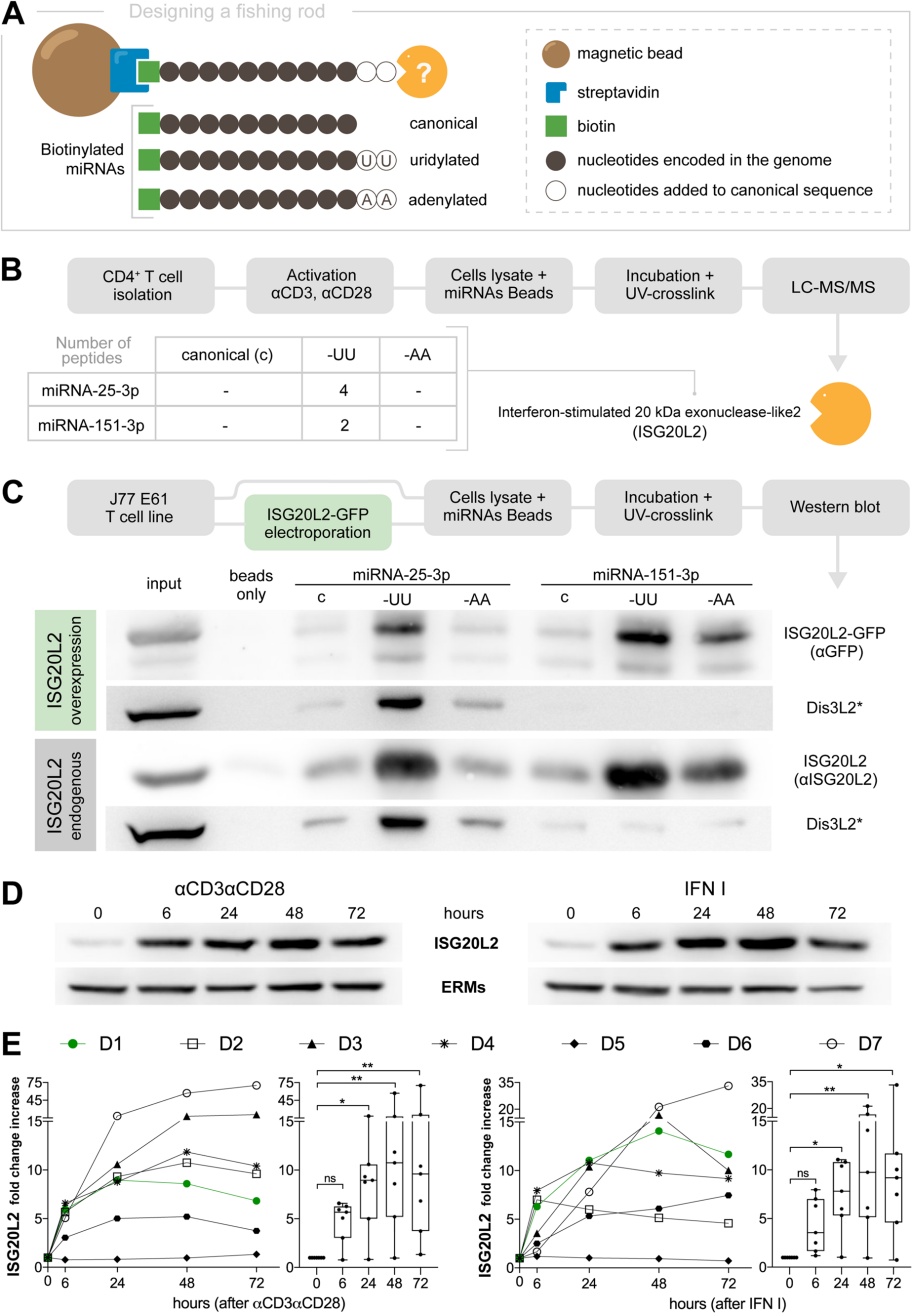
ISG20L2, a U′ exonuclease, upregulated in T cell stimulation. **A** Pull-down experiment design: biotinylated miRNAs either in their canonical sequence or with a 3′ addition of two uridines or two adenines were incubated first with T cell lysates. Thereafter, UV-crosslink to maintain RNA–protein interactions was performed, and streptavidin beads were added and recovered for analyses. **B** Amount of ISG20L2 peptides identified through mass spectrometry using a lysate of activated CD4 + T cells. **C** Western blot analyses showing ISG20L2 preferential interaction with uridylated miRNAs performing the pull-down experiment in J77 either relying on ISG20L2 GFP overexpression (top) or ISG20L2 endogenous expression (bottom). ISG20L2 overexpression images (top) show blots with anti-GFP and with anti-Dis3L2 antibodies. ISG20L2 endogenous images (bottom) show blots with anti-ISG20L2 and with anti-Dis3L2. Endogenous Dis3L2, an exonuclease described to degrade uridylated miRNAs, is shown as a positive control of U-interaction. **D**, **E** CD4 + resting T cells were isolated from healthy human donors and stimulated either with αCD3αCD28 antibodies (left panels) or with IFN I (right panels). Cells were incubated for 6 to 72 h and analysed by western blot. **D** Western blot images showing ISG20L2 and ERMs control expression, obtained from a representative donor (D1). **E** ISG20L2 fold change increment taking 0 h as a reference, measured for seven donors. Boxes extend from 25 to 75th percentiles with middle line at median and whiskers from the smallest to the largest value. Statistical analysis: Kruskal–Wallis test, Dunn’s multiple comparisons test [*p-value < 0.05, **p-value < 0.01, *ns* non-significant]

In order to identify enzymes which could be responsible for degradation of uridylated miRNAs after T cell activation, we designed a specific affinity-purification strategy. Three miRNA isoforms or isomiRs (canonical or modified by addition of two non-templated residues at the 3′ end, either adenines or uridines) of two different biotinylated miRNAs (miR-25-3p and miR-151a-3p) were bound to streptavidin magnetic beads and incubated with a lysate of activated CD4 + T cells; the miRNA-bound proteins were then identified by high-throughput liquid chromatography in tandem with mass spectrometry (LC–MS/MS) (Fig. 1A). Several peptides from ISG20L2 (Interferon-stimulated 20 kDa exonuclease-like 2) were identified specifically bound to uridylated miRNAs (Fig. 1B). These results were confirmed in the Jurkat (J77 E6.1) T cell line by western blot analysis, detecting overexpressed ISG20L2-GFP (blotting with αGFP antibody) and analyzing the dynamics of endogenous ISG20L2 (blotting with αISG20L2 antibody) (Fig. 1C). In both cases, ISG20L2 exhibited a higher affinity for uridylated miRNAs than for their canonical or adenylated forms. Interestingly, ISG20L2 interaction with miR-151a-3p-AA was higher than with the canonical miR-151a-3p, while its interaction with miR-25-3p-AA and its canonical form were not significantly different. These data point to a strong capacity of non-templated nucleotide additions to the miRNAs 3′ end to promote ISG20L2 target recognition, but also to a role of the miRNA sequence itself in determining this interaction. The substrate sequence seems to be particularly relevant also for Dis3L2 exonuclease, which was used as a positive control of known capacity to interact with uridylated substrates [8–13, 31]. Dis3L2 interacted preferentially with uridylated miRNA-25-3p, but barely recognized miR-151a-3p in any of its isoforms (Fig. 1C).

ISG20L2 protein expression kinetics was assessed by western blot in primary human CD4 + T cells from healthy donors stimulated either with αCD3αCD28 or with type I IFN (since ISG20L2 belongs to the family of IFN stimulated genes) for 6–72 h. A representative donor (D1) is shown in Fig. 1D, together with ISG20L2 expression kinetics from seven donors (Fig. 1E). ISG20L2 was upregulated after activation in every donor, except for D5, which exhibited higher ISG20L2 basal levels maintained through stimulation.

### ISG20L2 degrades miRNAs with a slight preference for uridylated substrates

**Fig. 2:**
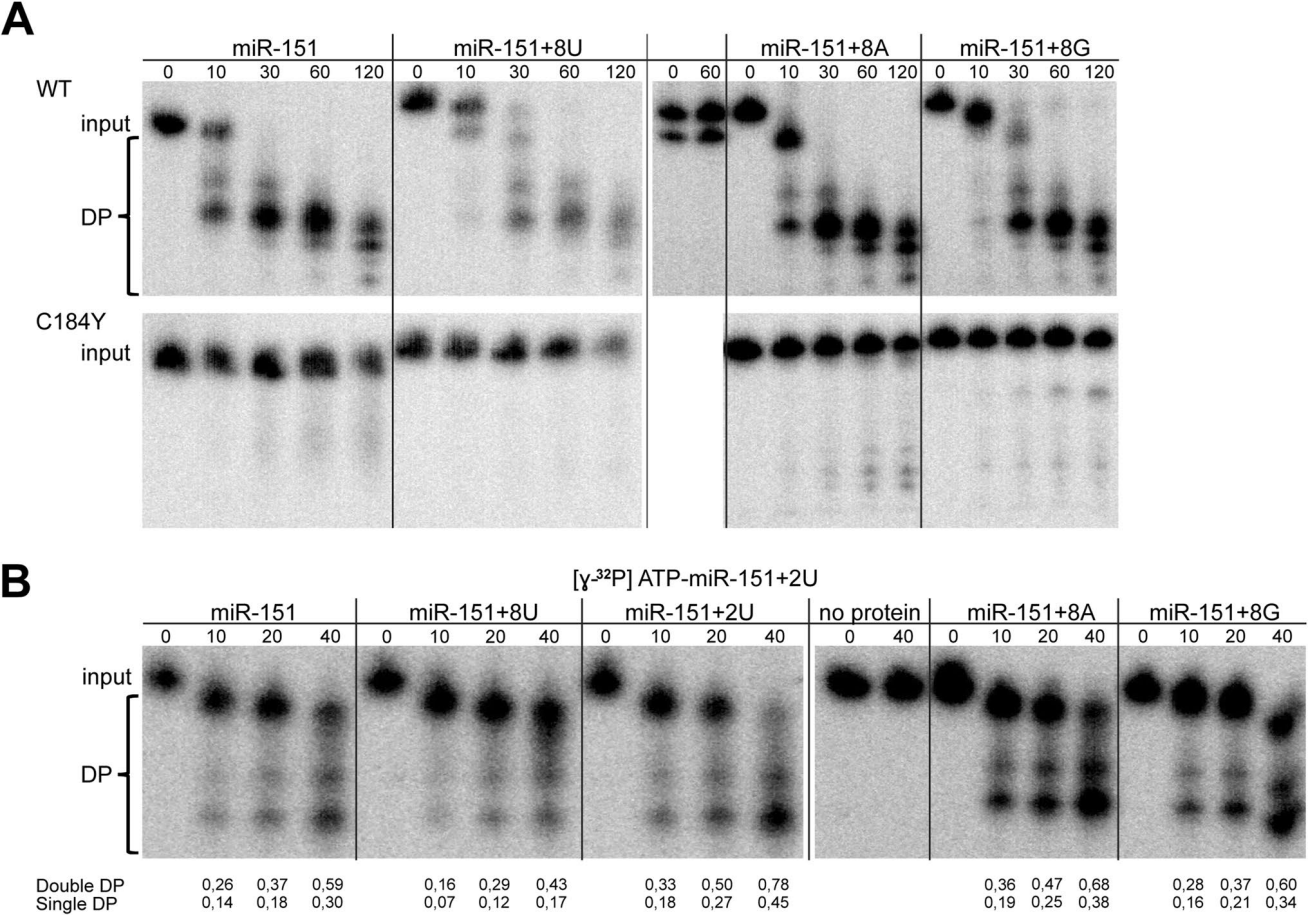
ISG20L2 displays degradation activity in vitro on miR-151a-3p bearing different 3′ terminal modifications. **A** ISG20L2 degradation activity on canonical miR-151a-3p and 3′ extended forms with additional 8 nt oligo(U), oligo(A) and oligo(G) 3′ extensions, respectively. RNA substrates labeled on 5′ end by γ-^32^P-ATP and 2 × excess of unlabeled RNA, were incubated with the recombinant purified wild type (WT) or with the catalytical mutant (C184Y) ISG20L2 for the indicated times. No protein mix is a control for unspecific degradation where RNA was incubated in the sample buffer for 30 min. RNAs were resolved by denaturing 20% gel electrophoresis and signals were detected by autoradiography. Input is the intact miR-151a-3p, DP are degradation products **B** 5′ end labeled miR-151a-3p possessing two additional UMP residues at its 3′ end was incubated with rISG20L2 in the presence of 10 × excess of unlabeled versions of miR-151a-3p with different 3′ terminal modifications (+ 8U, + 2U, + 8A, + 8G). The reactions and samples were processed as described in A. Quantifications indicate: single DP [lower degradation product signal (lower DP)/whole lane signal] and double DP [(lower + higher DP signal)/whole lane signal].

ISG20L2 catalytic activity was evaluated by in vitro degradation assays with different isoforms of miR-151a-3p and purified ISG20L2 protein. WT ISG20L2 displayed exoribonucleolytic activity on miR-151a-3p with and without 8 nt extensions at their 3′ end (Fig. 2A upper panel). No activity was observed with the recombinant C184Y ISG20L2 mutant (Fig. 2A, lower panel) confirming the functional relevance of C148 in the catalytic domain. The enzyme displayed rather processive activity leaving 4–5 nt degradation end product. To further assess whether addition of different homogeneous tails might affect ISG20L2 activity we incubated ISG20L2 with radioactively labeled miR-151a-3p + 2U and used differently tailed forms of miR-151a-3p in a 10 × molar excess. We observed that reactions with excess cold miR-151a-3p + 8U RNA showed the strongest inhibition on miR-151a-3p + 2U degradation monitored by the ratio of the accumulation of the degradation products over time (Fig. 2B). In summary these results show that ISG20L2 degrades miRNAs in vitro with a slight preference for U-tailed miRNA molecules.

### RNA and small RNA remodelling upon ISG20L2 silencing

**Fig. 3:**
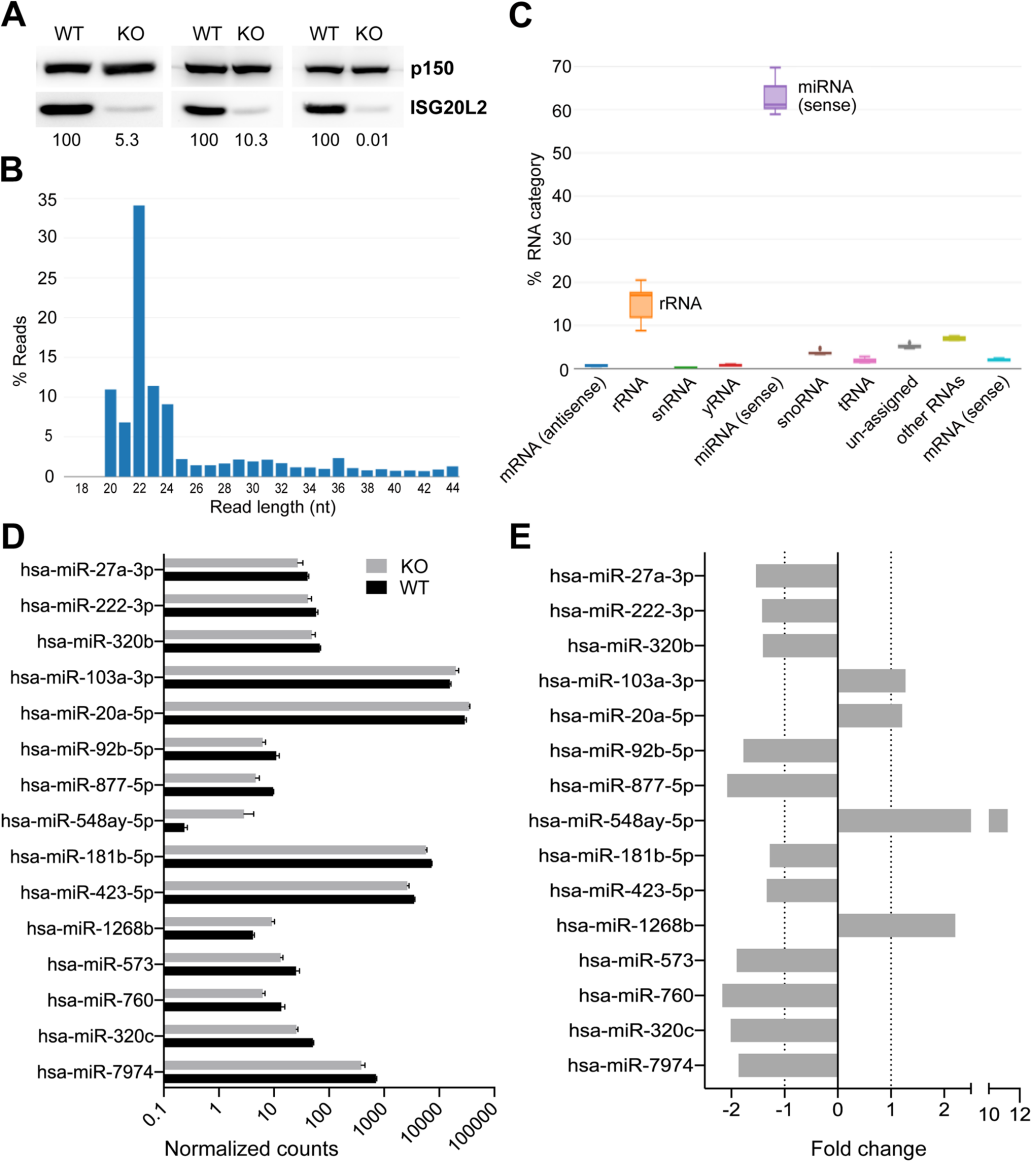
Small RNA sequencing of WT and KO ISG20L2 J77 cells. **A** ISG20L2 expression in sequenced samples (72 h after CRISPR-Cas9 plasmid electroporation). **B** Read length distribution in small RNAseq data for a representative sample. **C** Abundance of specific RNA types. **D** Normalized total counts per million (CPMs) detected for the 15 miRNAs with differential expression (log scale). **E** Limma estimated fold change for the 4 miRNAs upregulated in ISG20L2 knockout, and the 11 downregulated (15 miRNAs associated to Benjamini–Hochberg adjusted *p*-value < 0.1)

In order to gain a better understanding of the ISG20L2 targets in T cells, an NGS experiment was carried out to assess whether specific targets would be accumulated in the absence of the exoribonuclease. Three independent experiments were performed in J77 cells where ISG20L2 was deleted by CRISPR-Cas9 by at least 90% at protein level (Fig. 3A). Both small RNAs and mRNAs were sequenced, in an attempt to cover a wider range of potential substrates for the exonuclease ISG20L2.

RNAseq on small RNA was performed preparing libraries with a larger size than usual for this kind of sequencing, with the aim of detecting differential expression in other small RNA species apart from miRNAs. For this reason, the read length detected varies between 20 and 44 nt, although it has a clear peak around the usual miRNA size (22nt) (Fig. 3B). In fact, the presence of other small RNAs was very limited (Fig. 3C). A total of 597 miRNAs were detected in our samples, four of which: miR-92a-3p, miR-7-5p, miR-148a-3p and let-7f-5p accounted for almost 50% of the normalized counts (data not shown). Although we expected a global accumulation of specific targets in the absence of ISG20L2, only 15 miRNAs were found to be differentially expressed (adjusted *p*-value < 0.10): 4 upregulated and 11 downregulated in ISG20L2 knockout samples compared to control (Fig. 3D, E). Most of them with a modest fold change (around 1.2–2.2, in absolute value), except for miR-548ay-5p (11.2x) which was barely detected in control samples. Five differentially expressed miRNAs were detected with counts per million (CPMs) over 500, while the other ten did not sum more than 70 CPM on average.

**Fig. 4:**
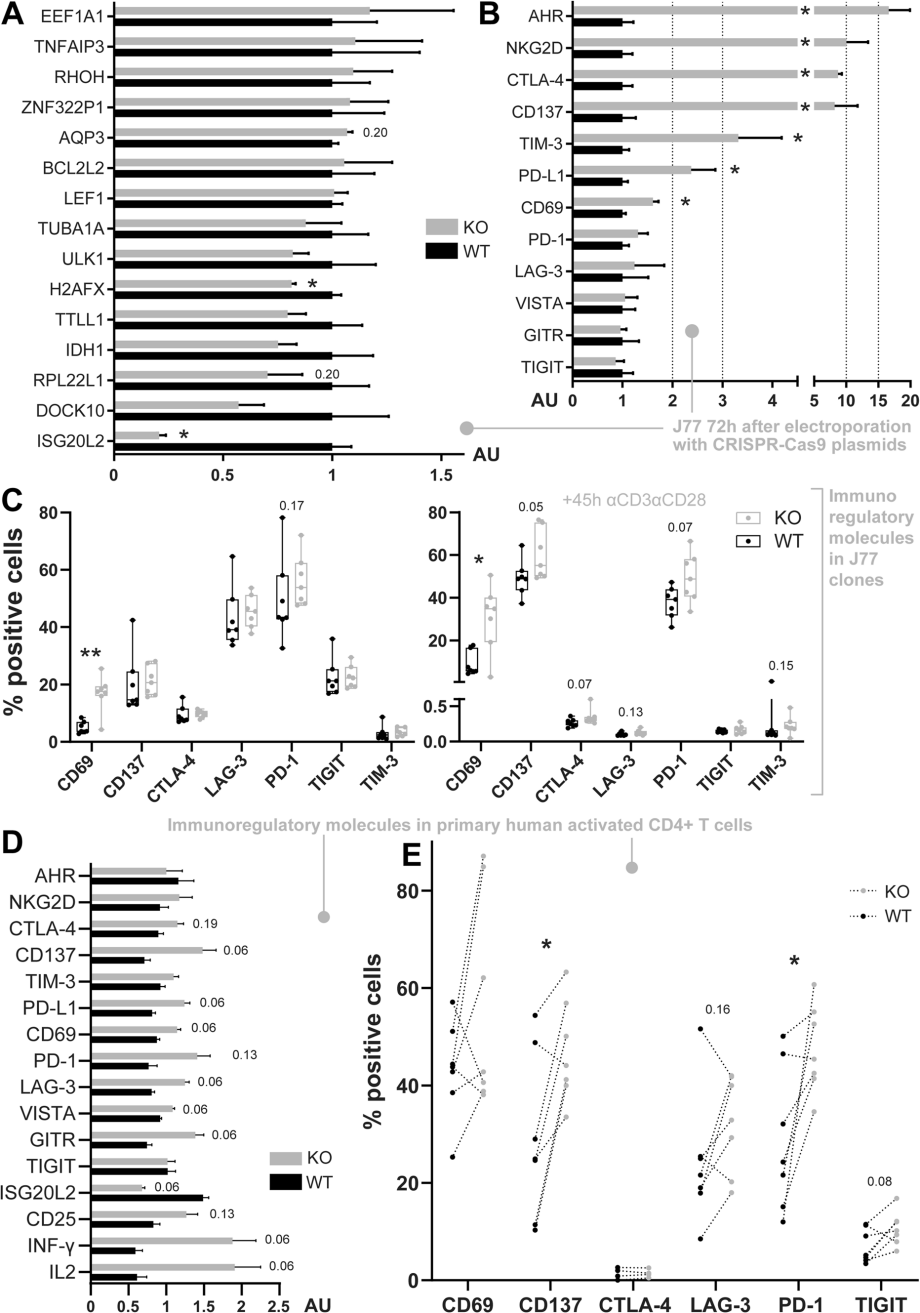
Control of immunoregulatory molecules by ISG20L2. **A**, **B** mRNA levels in J77 cells 72 h after electroporation with CRISPR-Cas9 plasmid control (Ctrl) or CRISPR-Cas9 plasmid targeting ISG20L2 (knockout, KO). **A** qPCR analysis of molecules detected with low p-adjusted values for RNAseq differential expression. **B** qPCR analysis of immunoregulatory molecules detected to be upregulated by RNAseq (CD69), relevant in IPA analysis (AHR, NKG2D, CTLA-4) and others. **C** Flow cytometry analysis, showing the percentage of cells expressing surface immunoregulatory molecules in resting (left) and activated (right) clones (WT and KO ISG20L2 established cell lines). **D** mRNA levels of immunoregulatory molecules and other molecules related with T cell activation in stimulated human primary CD4 + T cells. **E** Flow cytometry assessment of immunoregulatory molecules on the surface of activated human primary CD4 + T cells (data from cells obtained from the same donor is connected with a dotted line). **A**, **B**, **D** qPCR values are represented as mean ± SEM, analysed with Mann–Whitney statistical test. **C** Data are represented with box and whiskers (box includes 25th to 75th percentiles with middle line at median and whiskers from the smallest to the largest value). Data in C have been analysed with Mann–Whitney and paired data in E with Wilcoxon text. *p*-values 0.05–0.2 are indicated and statistical significance is depicted as * < 0.05, **0.01

Analyses of sequenced RNA comparing WT and ISG20L2 KO cells, revealed three differentially expressed genes with an adjusted *p*-value < 0.1: RPL22L1(fold change: − 1.30X), EEF1A1 (1.10X) and the knocked out ISG20L2 (− 1.56x) (Supplementary Table 1). Other genes with lower *p*-values detected for differential expression comprise a series of ribosomal proteins, lncRNAs, and certain molecules related with the immune response, such as CD69 (a marker of activation and immunoregulator [32]), LEF1 (transcription factor which binds to the TCR enhancer [33]), RHOH (a regulator of integrin LFA-1 binding avidity [34, 35]) or AQP3 (a modulator of T cell migration toward cytokines [36]). Among the molecules with lowest *p*-values we could confirm by qPCR ISG20L2 silencing, H2AFX downregulation and CD69 upregulation (Fig. 4A, B). However, RPL22L1 downregulation and EEF1A1 upregulation were not significant by qPCR (Fig. 4A). Several lncRNA, with low *p*-value or high fold change for differential expression, were also selected for further qPCR assessment without finding significant differences (data not shown).

### Control of immunoregulatory molecules by ISG20L2

Unbiased Ingenuity Pathway Analysis (IPA) of RNAseq data highlighted the relevance of ISG20L2 in the eukaryotic initiation factor-2 (EIF2) signalling pathway, involved in translation initiation; and in molecular and cellular functions related with RNA damage and repair, and protein synthesis (Supplementary Fig. 2). IPA also pointed to NKG2D and CTLA-4 as upstream regulators, targeting CD69 and LEF1, and CD69 and TENT5C, respectively (in core analysis of dataset with adj. *p*-values < 0.30); and to Aryl Hydrocarbon Receptor (AHR) signalling pathway as one of the significantly affected canonical pathways. Since CD69 was upregulated and AHR, CTLA-4 and NKG2D could be dysregulated upon ISG20L2 silencing, these and other molecules also known for their immunoregulatory function were evaluated. Interestingly, upregulation of several immunoregulatory molecules (AHR, NKG2D, CTLA-4, CD137, TIM-3 and PD-L1) besides CD69, was detected in ISG20L2 knockout cells (72 h after electroporation) (Fig. 4B). AHR, NKG2D, CTLA-4 and CD137 had been excluded from the RNAseq differential expression analysis because their expression was not detected over the established threshold (1 CPM in at least 3 samples). Most likely, mRNAseq sensitivity, lower than that of qPCR analysis, is not enough to detect the changes in these molecules at the level they are expressed in this context. Moreover, *CD69, CD137, CTLA-4 and PD-1* were also expressed at higher levels on ISG20L2 KO J77 activated clones (established cell lines) with a *p*-value < 0.1 (Fig. 4C).

ISG20L2 modulation of immunoregulatory molecules was also validated in human primary CD4 + Tcells. Expression of *CD137, PD-L1, CD69, LAG-3, VISTA, GITR, IFN-γ* and *IL2* genes was increased in ISG20L2 knockout cells compared to control after 72 h of reactivation (*p*-value < 0.10) (Fig. 4D). Surface expression of several immunoregulatory proteins was also assessed by flow cytometry after 48 h of reactivation, detecting a significantly higher percentage of cells expressing CD137 and PD-1, and an increase trend for molecules such CD69, LAG-3 and TIGIT in ISG20L2 KO cells(Fig. 4E).

### ISG20L2 controls CD69 and CD25 expression and limits IL2 production

In order to gain an understanding of ISG20L2 function in T cells, a set of experiments were performed comparing ISG20L2 KO with WT J77 T clones (established cell lines). ISG20L2 KO cells proliferation was significantly delayed compared to control cells (Supplementary Fig. 3A, B). To assess whether ISG20L2 had an effect on cell viability and apoptosis, Annexin V (Supplementary Fig. 4A, B) and cleaved Caspase-3 expression (Supplementary Fig. 4C, D) were evaluated after PMA and ionomycin stimulation. A higher percentage of apoptotic (Cleaved Caspase +) cells at 24 h and dead cells at 48 h (Annexin V staining) was detected within ISG20L2 KO cells. However, we did not find any significant difference in the percentage of live cells, therefore ISG20L2 does not seem an essential factor for cell survival (Supplementary Fig. 4A, C).

**Fig. 5:**
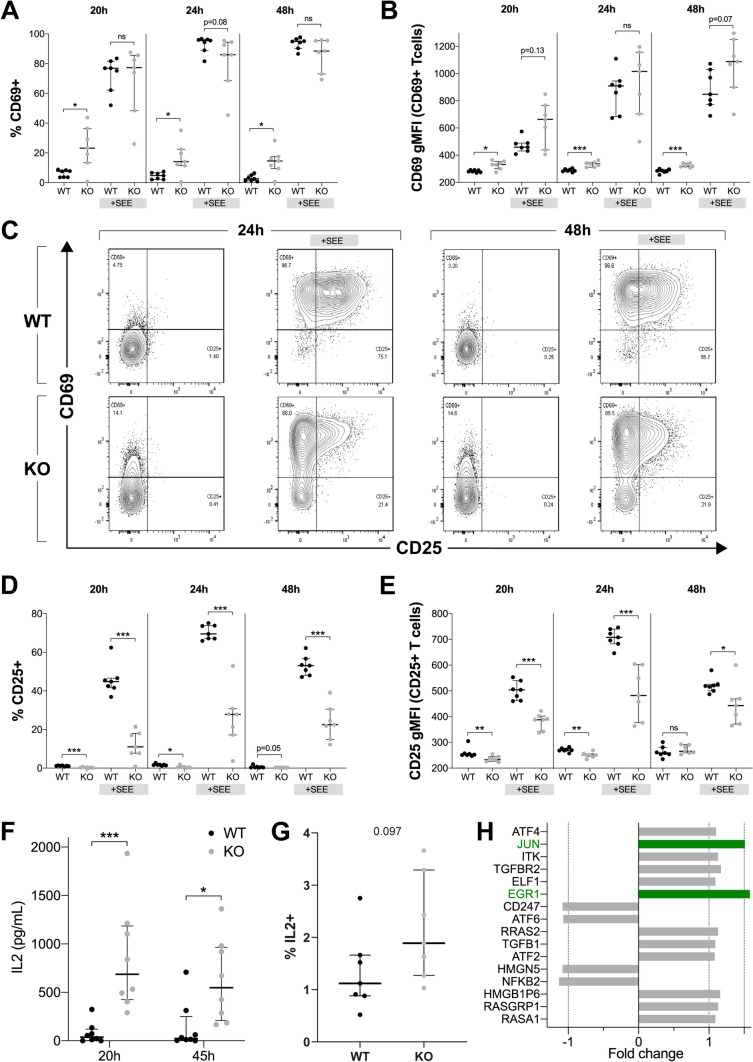
ISG20L2 controls CD69 and CD25 expression and limits IL2 production. WT or ISG20L2 KO T cell clones were cultured with Raji cells at 1:1 ratio (24 and 48 h) and 10:1 (20 h). Propidium Iodide was used to remove death cells, and CD19 to exclude B cells. **A** Percentage of CD69 + T cells at 20, 24, and 48 h, cultured with or without SEE. **B** Geometric Mean Fluorescence Intensity (gMFI) CD69 + T cells. **C** Representative WT and KO plots. **D** Percentage of CD25 + T cells at 20, 24, and 48 h, cultured with or without SEE. **E** Geometric Mean Fluorescence Intensity (gMFI) of CD25 + T cells. **F** ELISA IL2 release analysis after 20 or 45 h of PMA and ionomycin treatment in WT and ISG20L2 KO clones. **G** IL2 intracellular staining of WT and ISG20L2 clones stimulated with PMA and ionomycin for 20 h. **A**, **B**, **D**–**G** Each dot represents an individual clone, median and interquartile range are plotted in every graph. Mann–Whitney test was applied to assess statistical significance, which is indicated as * < 0.05, ** < 0.01, *** < 0.001, and *p*-values 0.05–0.2 are included as text. **H** Molecules detected in mRNAseq as differentially expressed upon ISG20L2 silencing, which could be involved in regulation of IL2 expression (adj. *p*-value < 0.5, molecules with lower *p*-values are at the top of the graph). Green bars correspond to molecules with a Fold change > 1.5 in KO samples

To assess T cell activation during antigen-driven intercellular interaction ISG20L2 KO J77 cells were co-cultured with Raji cells as Antigen Presenting Cell (APC) in the presence or absence of Super Antigen E (SEE). CD69 and CD25 activation markers were evaluated by flow cytometry (Fig. 5A–E). In basal conditions, ISG20L2 KO T cells present both higher CD69 surface expression (Fig. 5B) and higher percentage of CD69 + cells than WT (Fig. 4C, Fig. 5A, C). However, both WT and KO cells upregulate CD69 surface expression upon stimulation with Raji-SEE at similar levels. On the other hand, ISG20L2 KO cells showed reduced CD25 expression both in basal conditions and after activation (Fig. 5C–E).

IL2 production of WT and ISG20L2 KO clones was assessed after stimulation with PMA and ionomycin, and measured by ELISA (Fig. 5F) and flow cytometry (Fig. 5G). Since we detected a higher IL2 secretion in KO clones, we revisited the mRNAseq data comparing WT and ISG20L2-silenced cells to search for potential factors involved in IL2 expression. Within the list of potential candidates, JUN and EGR-1, transcription factors involved in IL2 production, were upregulated in knockout samples and may account for the higher IL2 production (Fig. 5H).

### ISG20L2 reduces CD3 accumulation at immune synapse and limits MTOC translocation

**Fig. 6:**
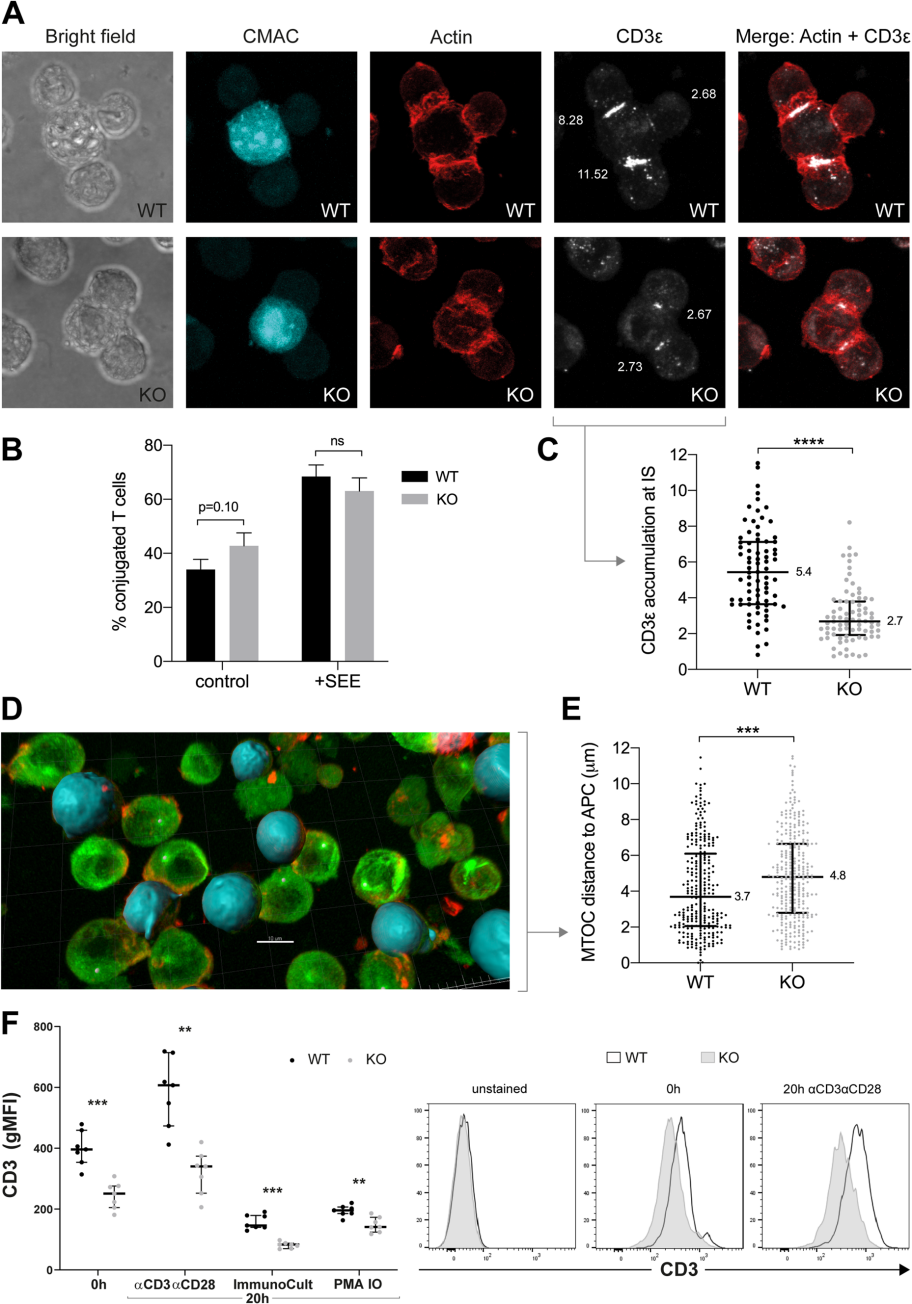
ISG20L2 is involved in CD3 accumulation at IS. **A** Representative Bright field, CMAC, Actin, CD3ε and merge (Actin + CD3ε) for WT and ISG20L2 KO T cells incubated with Raji (CMAC +) in the presence of SEE. **B** Percentage of conjugated T cells quantified as T cells in contact with B cells presenting enriched actin staining towards the B cell. **C** CD3ε accumulation at immune synapse (IS) quantified for WT and KO T cells in synaptic contact. CD3ε was not quantified in SEE absence, since it was not accumulated in contact areas. **D** Representative image for MTOC locations and Raji (APC) surfaces modelled with Imaris software. **E** MTOC distance to APC measured in μm. **F** Geometric Mean Fluorescence Intensity (gMFI) of CD3 surface expression on WT and KO clones in resting conditions and 20 h after activation with different stimuli (left). Representative KO and WT plots (right)

To assess whether synaptic contacts between T and B cells were occurring with ISG20L2 KO T cells in a similar way than with control cells, cell–cell conjugates were analysed by immunofluorescence. APC cells were previously stained with the cell-tracer CMAC, and co-cultured with T cells for 30 min. Conjugated T cells were quantified as T cells presenting an actin accumulation towards the contact site with APC cells (Fig. 6A, B). The proportion of T cells forming cell–cell conjugates was similar for WT and KO clones, both with and without SEE (Fig. 6B). CD3ε, a 20 kDa subunit of the TCR complex, is accumulated at the immune synapse (IS) (Fig. 6A). CD3ε accumulation ratio at the IS in ISG20L2 KO cells was halved compared to WT cells (Fig. 6C). Figure 6A shows a representative image with accumulation of CD3ε in KO cells at median values, and a diverse range of WT CD3ε increments at IS. Moreover, the distance from the synaptic T cell MTOC (microtubule-organizing centre) to the surface of the Raji B cell (modelled with Imaris software (Fig. 6D)) was also quantified, showing a slight increase in MTOC-APC distance in ISG20L2 KO cells (Fig. 6E), indicating an impaired MTOC translocation in the absence of ISG20L2.

The surface expression of CD3 on WT and KO clones was evaluated by flow cytometry both in resting state and upon different activation stimuli (Fig. 6F). CD3 abundance was always higher in ISG20L2 WT clones, indicating the decreased accumulation of CD3 at IS on ISG20L2 KO T cells could be due to a reduced availability of CD3 on their cell surface.

In order to evaluate how ISG20L2 could affect a series of organelles we electroporated WT and ISG20L2 KO clones with the ColorfulCell reporter plasmid and performed in vivo microscopy after TCR activation with αCD3αCD28 antibodies. Interestingly, nucleus and membrane sphericity were lower in WT clones (Supplementary Fig. 5), which could be the result of a better T cell polarization towards the activation stimulus on the plate surface. The distance between membrane and Golgi apparatus was also lower in WT clones (Supplementary Fig. 5), possibly also indicating an impaired polarization of Golgi upon activation in the absence of ISG20L2.

Altogether, these data support a novel immunoregulatory role of ISG20L2 in T cell activation, cytokine secretion and IS formation.

## Discussion

To gain insights into the enzymatic activities underlying miRNA remodelling during T cell activation, we searched for a potential enzyme responsible for the decreased expression of uridylated miRNAs following stimulation [6, 7]. Remarkably, an affinity-based identification strategy using uridylated miRNAs and lysates of activated T cells, allowed to identify ISG20L2, which was previously described as a 3′ to 5′ exoribonuclease [37]. Upon its initial identification in human nucleoli, ISG20L2 was associated with a function in ribosome biogenesis, specifically in the processing of the 12S precursor rRNA (a component of 5.8S rRNA). However, a possible additional role was already suggested due to its interaction with partners involved in mRNA splicing, exporting or trafficking [37].

In our experimental setting this enzyme could stabilize an interaction with miRNAs due to the presence of only two additional uridines at the 3′ end. Other factors apart from uridylation seem to be involved in ISG20L2 substrate recognition, since a different pattern of interaction was found comparing two distinct miRNAs, miR-25-3p and miR-151a-3p. Likewise, Dis3L2, an exonuclease known to preferentially degrade uridylated substrates [8–13, 31], binds to miR-25-3p isoforms in a similar way to ISG20L2, while it does not bind miR-151a-3p.

In agreement with the pull-down experiments, recombinant ISG20L2 was able to degrade miRNAs in vitro. While bacterial expression of the catalytically inactive form did not present any major problems, expression of ISG20L2 WT was extremely difficult. Overexpressing ISG20L2 WT in J77 T cells has also been highly challenging, achieving a very low percentage of positively electroporated cells (data not shown). Indeed, Zhou et al. analysing whether ISG20L2 and ISG20L1 shared ISG20 antiviral capacities, reported they did not succeed generating a Huh7.5 cell culture stably expressing ISG20L2 [38]. Therefore, ISG20L2 could be deleterious for bacterial and mammalian cells, due to its processive exoribonucleolytic activity, when expressed in large amounts or during long periods of time. In spite of the difficulties experienced in the production of recombinant ISG20L2, it was possible to obtain enough protein to perform in vitro RNA degradation assays which revealed that miRNAs are susceptible to ISG20L2 catalytic activity. ISG20L2 showed higher preference for oligoU tailed miR-151a-3p; however it could efficiently degrade miR-151a-3p independent of the 3′ end. It is therefore conceivable that additional cofactors or modifications regulate ISG20L2 function in vivo.

To further assess the target specificity of ISG20L2, we set to obtain an in-depth analysis of potential RNA substrates altered in ISG20L2 absence. Strikingly, only 15 miRNAs were detected as differentially expressed within small RNAs, specifically miR-548ay-5p was highly upregulated. Among the genes differentially expressed identified by RNAseq, only downregulation of H2AFX and upregulation of CD69 were confirmed by qPCR. Unexpectedly, IPA analyses led us to study immunoregulatory molecules. Expression of AHR, NKG2D, CTLA-4, CD137, TIM-3, PD-L1 and CD69 was upregulated upon ISG20L2 knockout when measured by qPCR. In ISG20L2 KO activated human primary CD4 + T cells, levels of the immunoregulatory genes *CTLA-4*, *CD137*, *PD-L1*, *PD-1*, *CD69*, *LAG-3*, *VISTA* and *GITR* were also upregulated with *p*-value < 0.2. Moreover, CD137 and PD-1 cell surface upregulation was confirmed by flow cytometry in activated primary cells. Altogether, these changes support that ISG20L2 downregulation increases the expression of molecules with T cell immunomodulatory capacity. Further research would be required to understand whether the regulation of these molecules by ISG20L2 occurs through miRNAs or other non-coding RNAs, directly on their mRNAs molecules, or with the participation of other intermediate players.

Given ISG20L2 upregulation upon T cell stimulation and its involvement in the regulation of a set of molecules capable of modulating immune responses, we investigated whether J77 ISG20L2 KO cells would present any functional impairment. Rather than a global effect on protein expression caused by a possibly defective ribosome translation machinery (ISG20L2 has been associated with ribosome biogenesis, particularly processing 12S precursor rRNA [37]), ISG20L2 KO cells showed a specific modulation of selected molecules, such as IL2 and its receptor CD25. IL2 expression is the result of a complex interaction between transcription factors which bind to the IL2 promoter region upon TCR stimulation. NFAT, AP-1, NF-kB, SP1/EGR-1 are some of the involved factors [39, 40]. Interestingly, JUN (AP-1 component) and EGR-1 which could be promoting IL2 expression in ISG20L2 KO cells, were upregulated in mRNAseq of ISG20L2 silenced J77 cells. CD137 upregulation in the absence of ISG20L2, could also account for the increased IL2 levels observed [41].

Moreover, KO clones exhibited a deficiency in immune synapse formation and dynamics, with a reduced MTOC and Golgi polarization towards the APC, and decreased CD3ε accumulation at the APC-contact site. TCR accumulation at the synapsis depends on the available TCR pool at the plasma membrane, which is reduced in KO cells, but also on vesicular trafficking [42]. In fact, a defective MTOC translocation towards the IS could be limiting CD3 accumulation and impairing cytokine production [43, 44]. An additional layer regulating CD3 surface levels occurs with its internalization upon antigen stimulation [45]. In this regard, reduced CD3 could by caused by a higher T cell activation in KO samples.

Although the function of ISG20L2 on T cell activation is far from being fully understood, our data point towards an enhanced activation status in the absence of ISG20L2. Higher levels of IL2, CD69, CTLA4, PD-1 and reduced CD3 expression in KO cells would support this view. Hence, this enzyme may function like a ‘break’ limiting T cell activation or promoting the contraction of the activation response.

Other ribonucleases have been shown to play key roles on immune system regulation. For instance, Eri1 which is also a 3′ to 5′ exoribonuclease associated with rRNA processing [46, 47], has been described to control the size of NK population and T cell viral responses [15]. Another example, is Regnase-1, an endonuclease which is involved in maintaining immune homeostasis, also avoiding spontaneous generation of effector CD4 + T cells [48, 49] and, therefore, regarded as a target to increase adoptive cell therapy efficiency against cancerous cells [50].

In summary, ISG20L2, an exonuclease with preferential specificity for uridylated miRNAs, is upregulated upon T cell activation and modulates several key aspects of T cell function, including activation, cytokine secretion and IS formation. ISG20L2 also acts as a negative regulator of immunoregulatory molecules such as CD137, PD1 or CTLA4. Although further research will be needed to uncover its mechanisms of action and to fully characterize its role in immune cells, our data already point to ISG20L2 as a novel RNA nuclease based immune regulator with a key role in T cell function.

### Supplementary Information

Below is the link to the electronic supplementary material.

Supplementary file1 (DOCX 2590 KB)

## Data Availability

GEO repository of sequencing data (access number pending).
