# Supplementary material for: ISG20L2: an RNA nuclease regulating T cell activation
Source: Cell Mol Life Sci. 2023 Aug 30;80(9):273. doi: 10.1007/s00018-023-04925-2 (PMC10468436; doi:10.1007/s00018-023-04925-2)
Supplement: Supplementary file 1 — Supplementary file1 (DOCX 2590 KB) [file 18_2023_4925_MOESM1_ESM.docx]

**
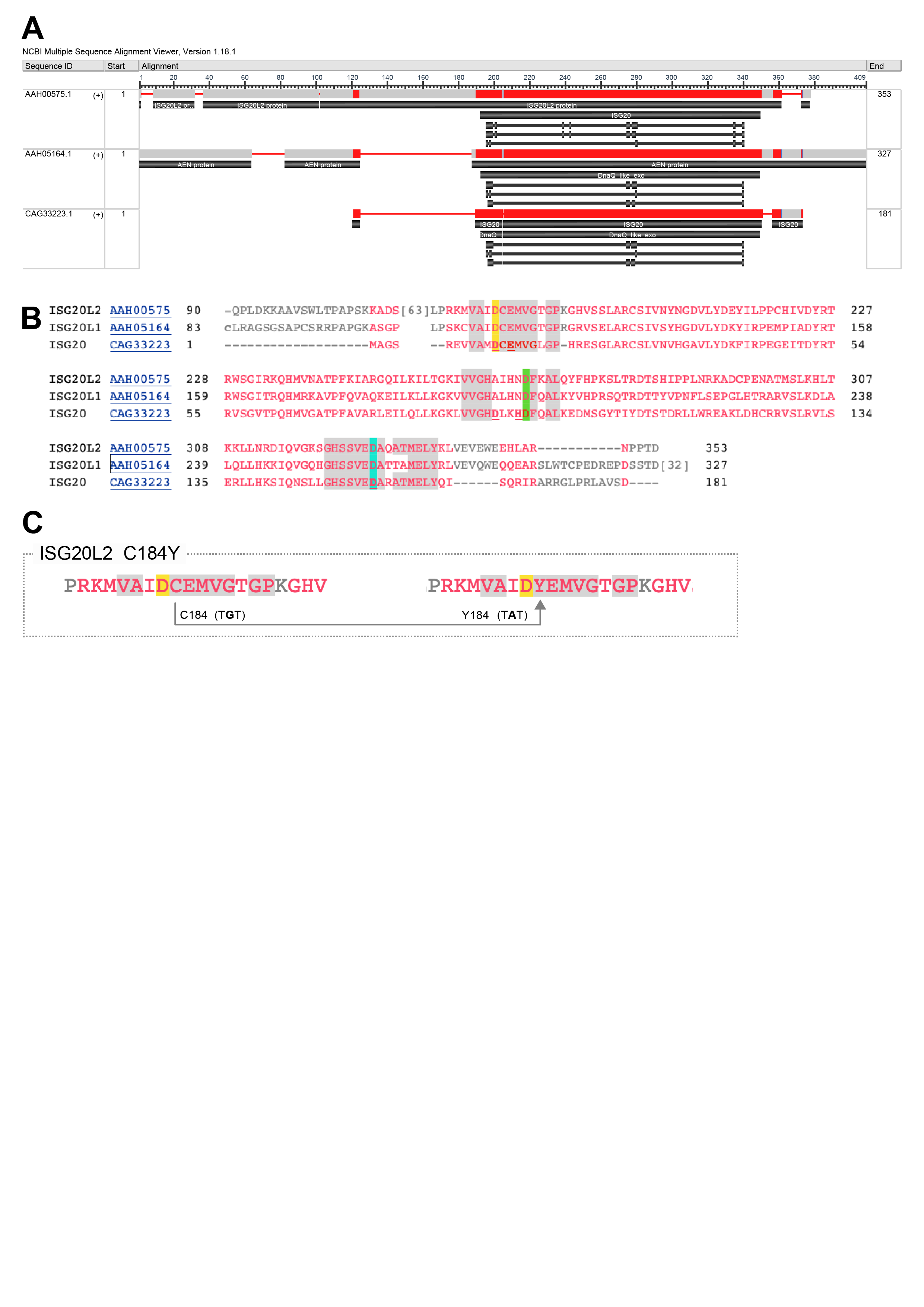
 Supplementary Fig. 1. Designing ISG20L2 catalytic mutant.**

(A) Alignment of conserved regions in the catalytic centre of ISG20L2, ISG20L1 (AEN) and ISG20 generated by COBALT (Constraint-based Multiple Alignment Tool). Red indicates highly conserved alignment columns (with no gaps) (column-method based on residue’s relative entropy threshold). DnaQ_like_exo region refers to DnaQ-like (or DEDD) 3’-5’ exonuclease domain superfamily. Regions identified without text included due to space limitations refer to active sites and substrate binding sites. (B) Sequence alignment of ISG20L2, ISG20L1 and ISG20. Bold and underlined residues have been described to be key sites for ISG20 catalytic activity. Aspartic (D) conserved residues are coloured in yellow, green and blue. (C) Mutations introduced to generate catalytic mutant C184Y. Cysteine in position 184 is replaced for a tyrosine through a one base nucleotide exchange (G to A) to generate C184Y mutant.

**
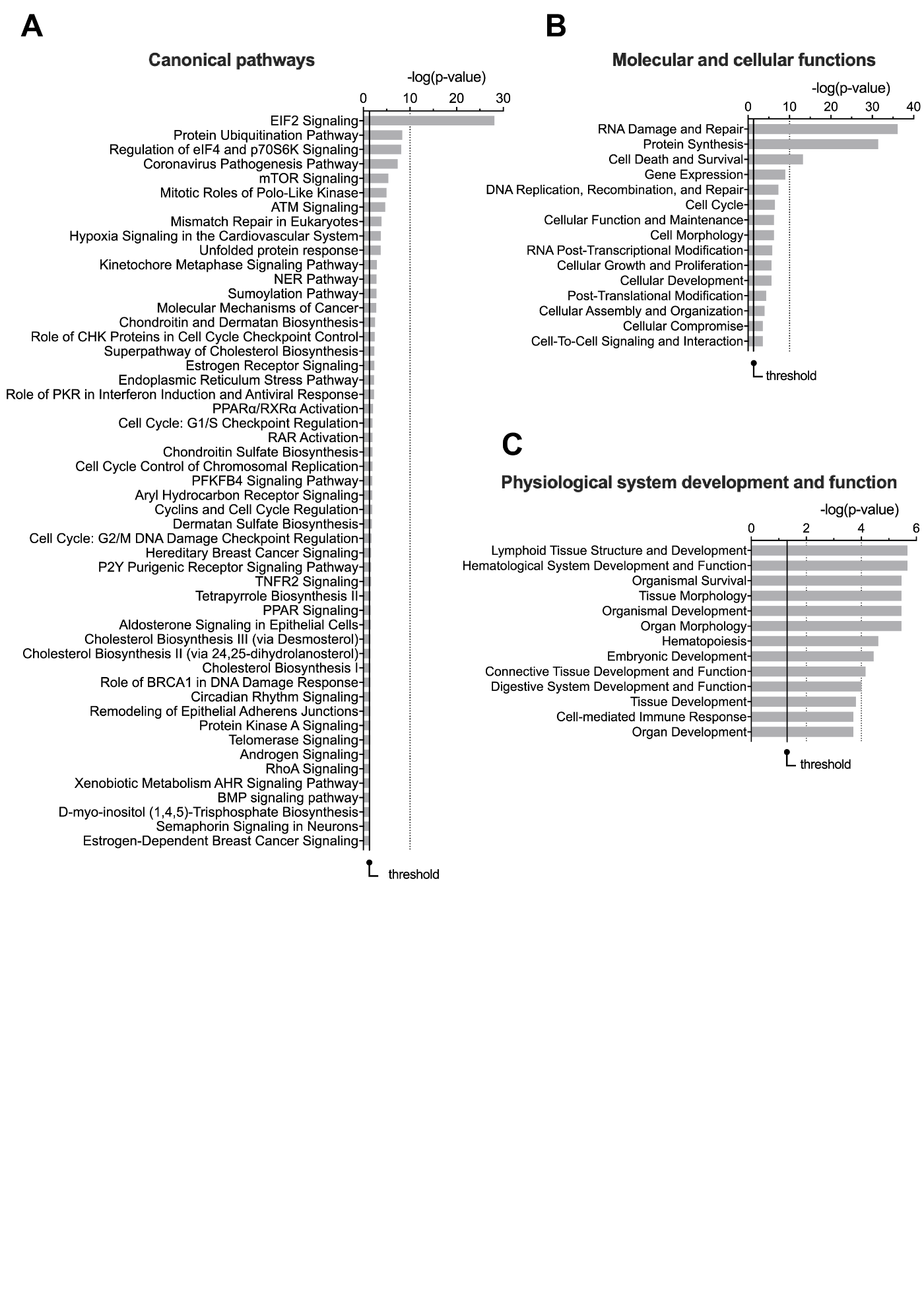
**

**Supplementary Fig. 2. ISG20L2 mRNAseq IPA core analysis.**

Ingenuity pathway analysis of mRNAseq molecules with differential expression between control and ISG20L2 knockout samples detected with an adjusted p-value<0.5 (p-value <0.05254), considering experimentally observed and predicted data, and direct and indirect relationships. (A) Canonical pathways. (B) Molecular and cellular functions. (C) Physiological system development and function.

**
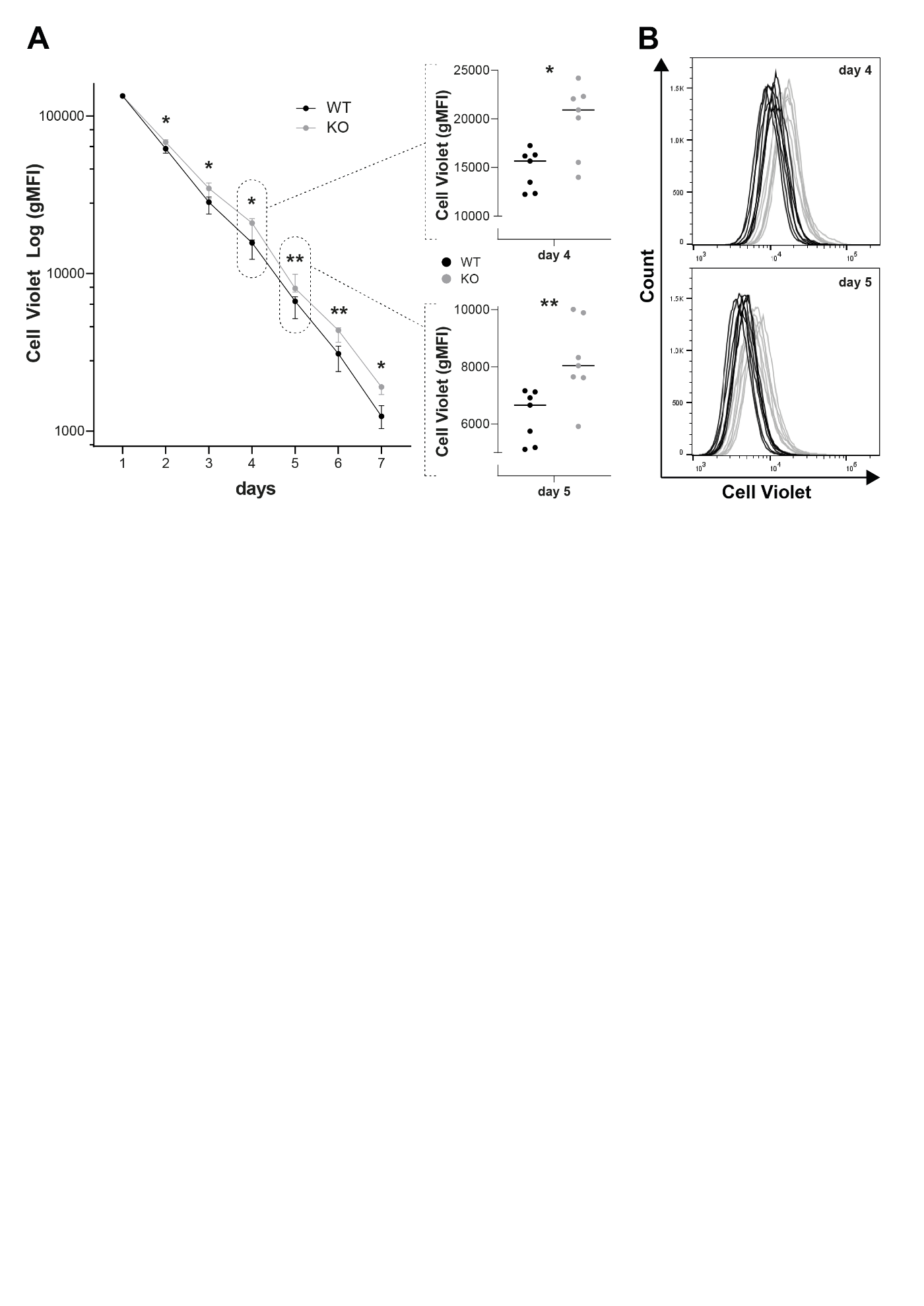
**

**Supplementary Fig. 3. ISG20L2 absence delays T cell proliferation.**

(A) WT and ISG20L2 KO T Cell Violet staining was followed for 7 days (left). Geometric Mean Fluorescence Intensity (gMFI) scatter dot plot included as an example for days 4 and 5 (right). (B) gMFI Cell Violet histogram for each WT (black) and KO (gray) clone at day 4 (top) and day 5 (bottom).

**
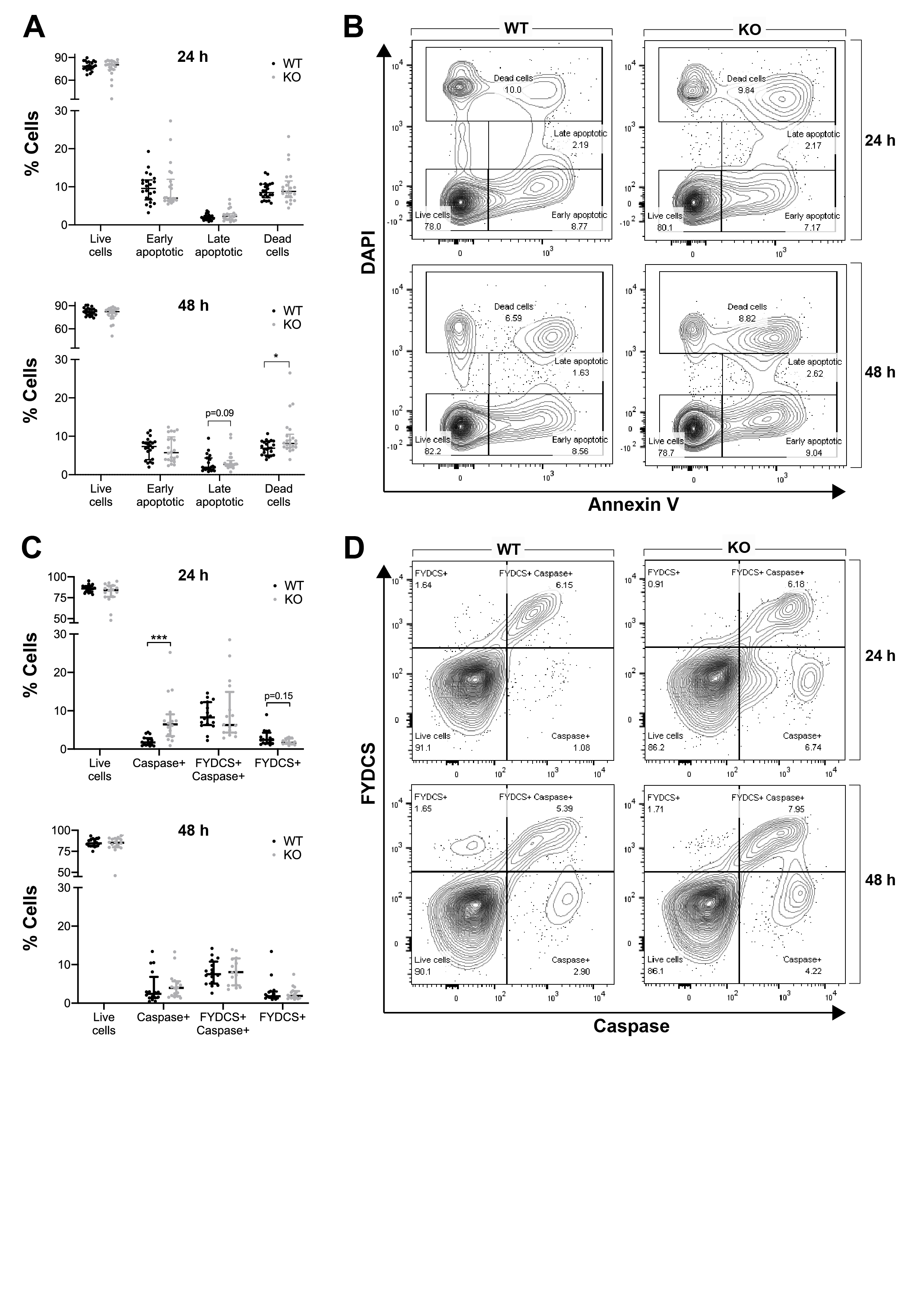
**

**Supplementary Fig. 4. ISG20L2 absence does not substantially affect cell viability.**

WT and ISG20L2 KO T cell clones were cultured with PMA and ionomycin for 24h and 48 h. (A,B) DAPI (cell viability dye) and Annexin V staining. (C,D) FYDCS (Fixable Yellow Dead Cell Stain) and Cleaved Caspase-3 intracellular staining. All plots show whole flow cytometry recorded events after a unique gating step excluding debris with very low side and forward scatter.

**
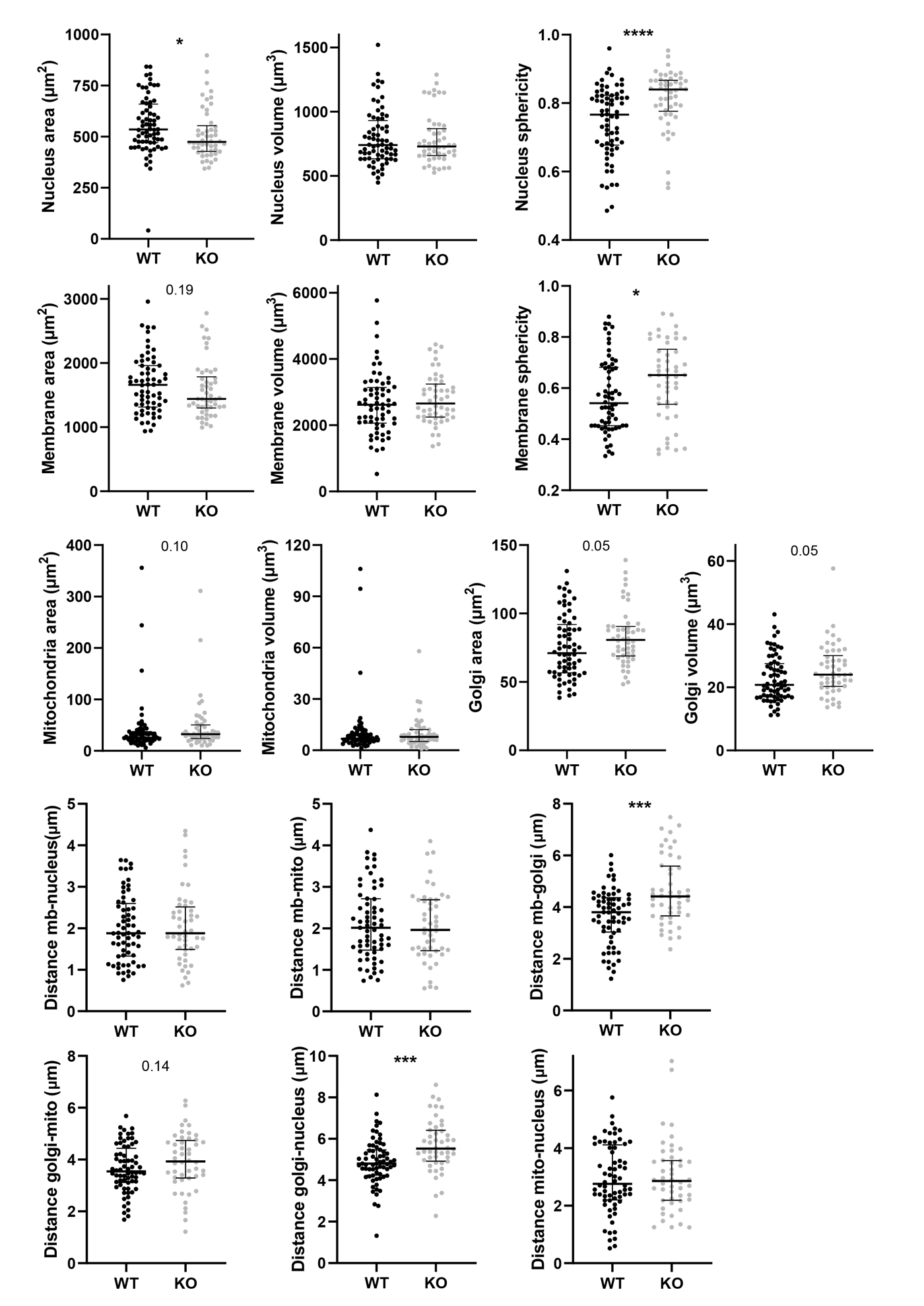
 Supplementary Fig. 5. Live imaging with ColorfulCell plasmid.**

WT and ISG20L2 KO T cell clones were electroporated with ColorfulCell reporter plasmid. Data shows Golgi, mitochondria, membrane and nucleus measures, and distances between organelles, calculated with Imaris software.


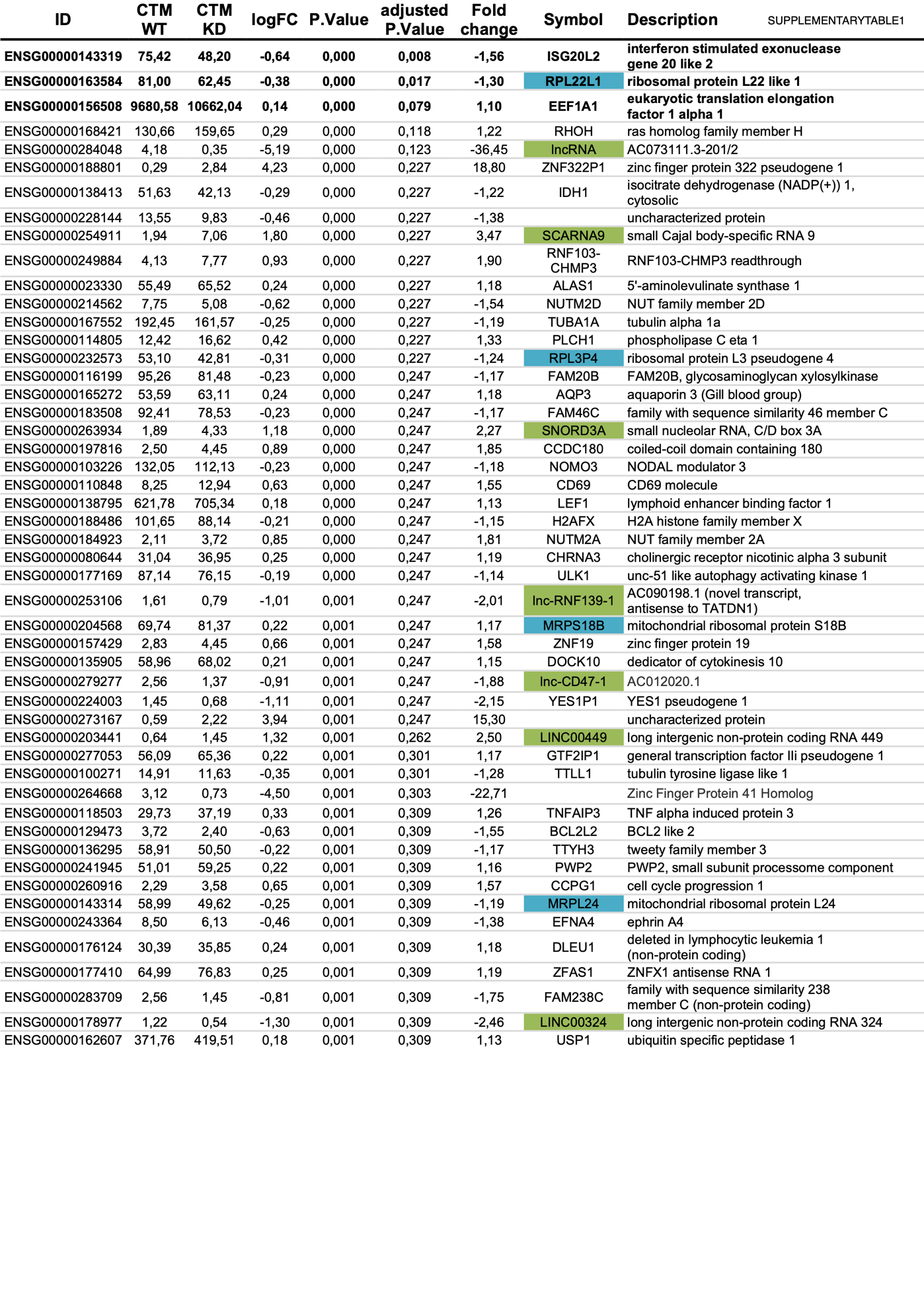


**Supplementary Table 1. mRNAseq ISG20L2 knockout vs control samples.**

mRNAseq molecules with the 50 lowest p-values for differential expression. Ribosomal proteins are marked in blue and lncRNAs, in green.
